# Priorities for Evidence-Based CKD Guidance Using Delphi Methods

**DOI:** 10.1016/j.ekir.2026.106573

**Published:** 2026-05-06

**Authors:** Brydee Cashmore, Martin Howell, Chandana Guha, Allison Jauré, Jonathan Craig, Rathika Krishnasamy, Vincent W. Lee, Nicole Scholes-Roberston, Amanda Sluiter, David J. Tunnicliffe, CARI Steering Committee and Cochrane Kidney and Transplant Management Committee, Bhadran Bose, Leanne Brown, Helen Coolican, Vanessa Cullen, Jeffrey Ha, Kelly Lambert, Casey Light, Min Jun, Thu Nguyen, Emmy O’Neill, Emily See, Carla Scuderi, Andrea Viecelli

**Affiliations:** 1 Faculty of Medicine and Health, Sydney School of Public Health, The University of Sydney, Sydney, New South Wales, Australia; 2 Centre for Kidney Research, The Children’s Hospital at Westmead, Westmead, New South Wales, Australia; 3 Faculty of Medicine and Health, Menzies Centre for Health Policy and Economics, The University of Sydney, Sydney, New South Wales, Australia; 4 College of Medicine and Public Health, Flinders University, Adelaide, South Australia, Australia; 5 Faculty of Medicine, Centre for Kidney Disease Research, The University of Queensland, Brisbane, Queensland, Australia; 6 Department of Nephrology, Sunshine Coast University Hospital, Birtinya, Queensland, Australia; 7 Faculty of Medicine and Health, Westmead Applied Research Centre, The University of Sydney, Sydney, New South Wales, Australia

**Keywords:** clinical practice guideline, consumer involvement, Delphi, kidney disease, patient-centered care, priority setting

## Abstract

**Introduction:**

Topic selection for systematic reviews and clinical practice guidelines often lacks transparent prioritization and meaningful community input. We aimed to identify shared priorities between people with lived experience of chronic kidney disease (CKD) and health-professionals to align evidence-based care with patient-clinician needs.

**Methods:**

We conducted a 3-round modified-Delphi survey across the following 4 modules: nondialysis CKD, peritoneal dialysis (PD), hemodialysis (HD), and transplantation. Round-1 participants rated (9-point Likert) and ranked (Best-Worst Scaling [BWS]) broad topics. Priority topics were refined into subtopics and rated in round-2; round-3 re-rated and ranked priority subtopics. Topic progression criteria included Likert thresholds, agreement (scores 7–9), and BWS scores (estimated via multinomial logistic regression), with data summarized descriptively (mean ± SD, % agreement).

**Results:**

Participation was 102 in round-1 (27% consumers) increasing to 613 in round-3 (71% consumers). Round-1 priority topics were as follows: Evaluation and management of CKD (8.09 ± 1.11, 93%), Infection and peritonitis management (PD; 7.98 ± 1.41, 88%), Cardiovascular disease management (HD; 7.96 ± 1.33, 89%), and Transplant rejection (8.36 ± 1.05, 96%). Across rounds 2 and 3, the prioritized subtopics were as follows: Treatment to slow CKD progression (6.37 ± 2.53, 56%), Maintenance of residual kidney function (PD; (7.19 ± 2.10, 96%), Volume control (HD; 7.15 ± 2.12, 75%), and Management of medication side-effects (transplantation; 6.65 ± 2.53, 65%). Where consumer-clinician differences emerged, consumers prioritized daily-life impacts (symptoms/side-effects, psychosocial support, infection prevention, and education), whereas clinicians emphasized care delivery (protocols, eligibility, and monitoring).

**Conclusion:**

This multistakeholder prioritization distilled broad kidney care domains into actionable subtopics, centered around slowing CKD progression and improving symptoms during kidney replacement therapy, providing targeted systematic reviews and guideline updates supporting shared decision-making in CKD.

## Graphical abstract

**Figure:**
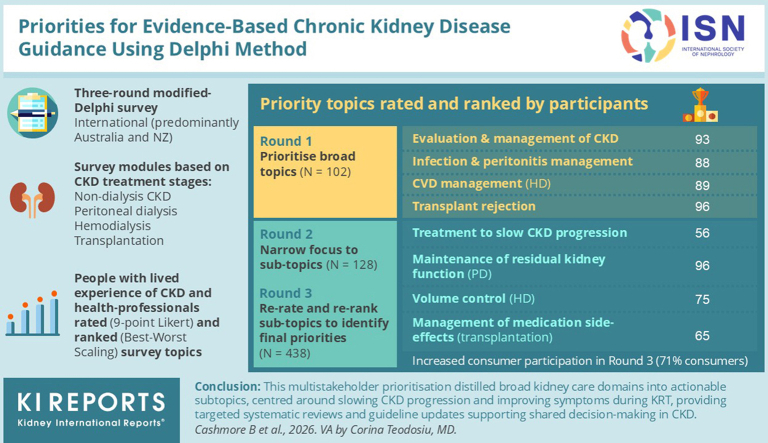

CKD is a global health burden, contributing to substantial morbidity, premature mortality, and healthcare costs.^1^^,^^2^ CKD progression profoundly impacts quality-of-life and brings complex physical, social, and psychological challenges. Kidney research has historically been underfunded relative to other medical specialties, limiting high-quality trials and slowing innovation in nephrology care.^3^, ^4^, ^5^ Recent therapeutic advances, however, offer promise for slowing CKD progression and preserving kidney function.^6^, ^7^, ^8^, ^9^, ^10^, ^11^ Translating these advances into routine care is essential to close the evidence-practice gap. Clinical practice guidelines (“guidelines”) are central to this translation, they rely on rigorous processes, including systematic reviews to synthesize evidence, as well as appraising acceptability and feasibility of recommendations in practice. Yet guideline development can be hampered by methodological inconsistency, limited stakeholder engagement, resource constraints, and slow update cycles, all of which impede timely implementation into clinical practice. With finite resources, numerous clinical questions compete for attention across the CKD stages and treatment modalities. Without coordinated prioritization and implementation strategies, even high-quality evidence may have limited impact.

As recommended by guideline development organisations,^12^^,^^13^ guidelines should be developed through consultation with clinicians and people with lived experience (consumers) to ensure their scope reflects shared needs. Limited engagement with the people the guidelines are intended to serve remains a barrier. Guideline development in CKD has traditionally been expert-led by panels in a “top-down” manner,^14^ which can inadvertently overlook the most pressing needs of patients.^5^^,^^15^, ^16^, ^17^ This may divert resources from high-priority areas and delay guidance where its most needed. Without structured, inclusive priority setting, the translational gap between evidence and practice is likely to persist.^15^

This study aimed to identify nephrology priority topics for systematic reviews and guidelines through multi-stakeholder engagement. By involving CKD consumers, clinicians, researchers, and policymakers, we sought to support shared decision-making and ensure that the scope of evidence-based guidance in CKD reflects what matters most to the community.

## Methods

**Figure 1:**
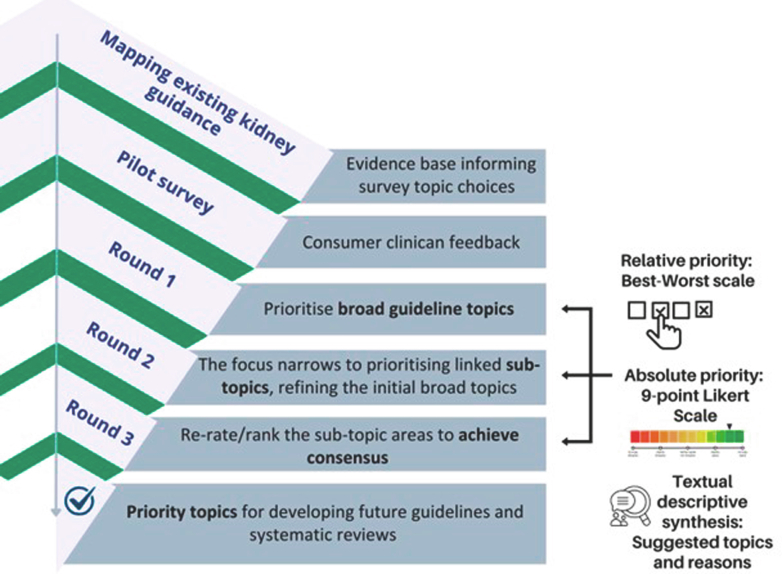
Prioritization process overview.

We conducted a 3-round modified-Delphi survey across multiple stakeholders (Figure 1), guided by a prespecified protocol (Supplementary File S1), and reported per Accurate Consensus Reporting Document checklist^18^ (Supplementary Table S1).

### Participants and Recruitment

Ethics approval was obtained from the University of Sydney (2023/HE000180). Participants received standardized information and provided informed consent online. Surveys were administered via Qualtrics, with responses deidentified and assigned unique participant codes. Consumers from Cochrane Kidney and Transplant and Caring for Australians and New Zealanders with kidney Impairment (CARI) guidelines contributed to all stages, including protocol development, survey design, and dissemination of findings.

Snowball sampling was used to recruit participants.^19^ Consumers (patients/caregivers) were recruited via consumer research networks including the Better Evidence and Translation – CKD , Kidney Health Australia, Polycystic Kidney Disease Australia, and approved social media channels. Health professionals were recruited via investigator networks and professional societies in Australian and New Zealand. Participants were reinvited by email for each round, and new participants could join later rounds to maximize inclusion. A gift card worth A$15 was offered to consumers after completing all rounds.

### Data Collection

We mapped English-language clinical practice guidelines to identify topics. This inventory included clinical practice guidelines from the CARI, Canadian Society of Nephrology, European Renal Association-European Renal Best Practice, Kidney Disease: Improving Global Outcomes, Kidney Disease Outcomes Quality Initiative, National Institute for Health and Care Excellence, International Society for PD, and the British Transplantation Society. A list of 20 topics per module, CKD not requiring dialysis (nondialysis CKD), PD, HD, and kidney transplantation (Transplant), was developed and subdivided into subtopics based on the clinical questions covered. The list was reviewed by nephrology health professionals, consumers, CARI Steering Committee, and Cochrane Kidney and Transplant Management Committee, to ensure comprehensiveness and clarity.

Related Cochrane reviews were identified from the Cochrane Database of Systematic Reviews and mapped to guideline subtopics. Links to these, along with links to current guidelines, were later embedded in the surveys to inform responses. Ongoing clinical trials were identified from ClinicalTrials.gov using relevant search terms of the population and interventions.

**Figure 2:**
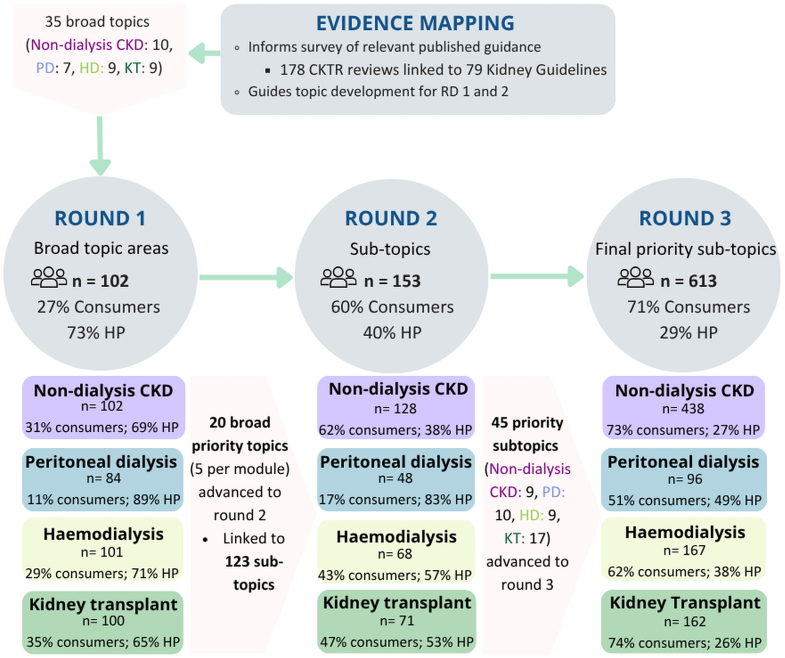
Overview of survey participants in each module across each survey round. ∗Dual-identity participants (consumer and health professional) were categorized as health professionals if they were a nephrologist, GP, or kidney specialist. All others were coded as consumers. N refers to number of participants. CKD, chronic kidney disease; CKTR, Cochrane Kidney and Transplant; HD, hemodialysis; HP, health professionals; KT, kidney transplantation; PD, peritoneal dialysis; RD, round.

After an online pilot survey incorporating consumer and clinician feedback, we launched an online 3-round modified-Delphi survey, informed by mapping and previous prioritization work in nephrology^16^^,^^20^, ^21^, ^22^ (Figure 2). Participants selected modules based on CKD treatment stage (nondialysis CKD, PD, HD, and Transplant) and rated topics and subtopics using 9-point Likert scales as follows: limited importance (score 1–3), important but not critical (score 4–6), critically important (score 7–9), and “unsure”^23^ and ranked them using Best-Worse Scaling (BWS) to evaluate the relative importance of topics.^23^^,^^24^ Each Likert topic was accompanied by a brief plain-language description and example subtopics where appropriate. Additional topics suggested by ≥ 10% of participants were included in the next round. Topics order was randomized to avoid ordering bias.

#### Round 1 Survey

Round 1 survey ran from December 2023 to April 2024, prioritizing broad topics using Likert scales and BWS, where participants identified the most and least important topics from sets of 4. BWS used a balanced incomplete block design with participants randomized to 1 of 6 blocks with 6 BWS choice tasks. Topics were identified as a priority if they met the following criteria: a mean Likert score ≥ 7.5 for health professionals and consumers, ≥ 70% agreement between consumer and health professionals,^25^ and from the BWS, a preference score ranked in the top 5 per module.

#### Round 2 Survey

Round 2 (May to July 2024) refined round 1 priorities by rating related subtopics. For example, the priority ‘cardiovascular disease in nondialysis CKD’ was unpacked into subtopics such as “Cardiovascular disease risk assessment,” “Lipids and triglycerides management,” “Heart failure treatment,” and “Renin-angiotensin system inhibition.” BWS was not included in round 2 to reduce survey fatigue, given the substantial number (*n* = 123) of subtopics presented. Subtopics progressed to round 3 if mean Likert score was ≥ 7 and ≥ 65% agreement. For transplant topics, thresholds were set higher ≥ 7.5 and ≥ 75%, because of higher scores compared with the other modules. Two overlapping round 2 transplant subtopics from different Likert lists were merged into “Ongoing management of immunosuppression” for round 3 to avoid duplicate representation in the BWS rankings.

#### Round 3 Survey

Round 3 ran from August to October 2024. Using the same methods as round 1, participants re-rated (Likert scales) and ranked (BWS) high priority subtopics from round 2. To facilitate informed decision-making, each module opened with aggregated, anonymized round-2 feedback (distributions by role, consumer vs. health professionals).

### Data Analysis

Consumers (patients and caregivers) were grouped for analysis. Dual-role participants (both a consumer and health professional) were classified as health professionals to avoid double-counting and distortion of estimates. Descriptive statistics were calculated and figures developed in R Studio (Posit PBC, Boston, MA).^26^ Topic agreement was assessed by calculating the percentage of participants who rated a topic as “critically important” (Likert 7–9). Unsure responses were excluded from mean Likert scores but included in denominators. BWS data was analyzed using multinomial logistic regression, NLOGIT (Econometric Software Inc., Plainview, NY)^27^), using the lowest-ranked topic as the reference. Regression coefficients (preference score) with 95% confidence intervals (CI) indicated relative importance and within-group variability. Prioritization thresholds used Likert distributions and BWS relative weights, with module-specific progression criteria set *post-hoc.*

Participants could provide free-text comments to support their ratings and could also suggest new topics. One reviewer (BC) conducted an initial textual descriptive synthesis, coding suggestions into distinct concepts and grouping into broader topics. This inductive approach enabled the identification of recurring priorities and emerging areas of interest. A second reviewer (DT) reviewed coded outputs, any disagreements were resolved through discussion. Suggestions informed new or under-represented topics for future rounds, and comments contextualized participants’ prioritization decisions.

## Results

Participation increased across rounds, with consumers representing 27% in round 1, 60% in round 2, and 71% in round 3, including more caregivers (41% of consumers). Figure 2 shows the breakdown of survey participants across modules. Round 1 participants were predominantly from Australia (89%), round 2 from Australia (60%) and the USA (23%), and round 3 mostly from Australia (83%).

Kidney transplant (65%) comprised the largest treatment modality reported in rounds 1 and 2, and HD the largest in the final round (Table 1). Among health professionals, nurses (round 1: 42%, round 2: 32%, and round 3: 24%); nephrologists (round 1: 25%, round 2: 29%, and round 3: 27%); allied health (round 1: 21%, round 2: 21%, and round 3: 15%); and researchers or policy makers (round 1: 38%, round 2: 21%, and round 3: 23%) were the most common, with > 80% reporting more than 10 years of nephrology experience (Table 2). Retention was 60% from round 1, and 54% from round 2. Indigenous representation increased from 7% in round 1 to 17% in the final round.

**Table 1:** Characteristics of patients/caregivers

Characteristic	Round 1, *n* (%) 27 participants	Round 2, *n* (%) 100 participants	Round 3, *n* (%) 439 participants
Participant type			
Patient	22 (81)	77 (77)	263 (59)
Caregiver	5 (19)	23 (23)	176 (41)
Age group (yrs)			
18–29	1 (4)	5 (5)	52 (12)
30–39	2 (7)	36 (36)	205 (47)
40–49	6 (22)	32 (32)	127 (29)
50–59	9 (33)	15 (15)	46 (11)
> 60	9 (34)	7 (7)	7 (2)
Not reported	0	5 (5)	0
Age at diagnosis (yrs) (patient only)			
< 18	3 (14)	5 (6)	13 (5)
18–30	3 (14)	17 (22)	114 (44)
31–40	6 (29)	12 (16)	89 (34)
41–50	7 (33)	20 (26)	39 (15)
51–60	1 (5)	2 (3)	4 (1)
> 60	1 (5)	1 (1)	1 (1)
Not reported	1 (5)	20 (26)	1 (1)
Cause of kidney disease (patient only)			
Diabetes mellitus	1 (5)	18 (23)	44 (17)
High blood pressure	0 (0)	9 (12)	0 (0)
Glomerular disease	9 (43)	18 (23)	123 (47)
Polycystic kidney disease	7 (33)	7 (9)	38 (15)
Other	2 (10)	3 (4)	20 (8)
Unknown	2 (10)	1 (1)	36 (14)
Not reported	1 (5)	21 (27)	0
Current treatment modality for CKD (patient only)			
Reduced kidney function	4 (18)	5 (6)	47 (18)
Hemodialysis	2 (10)	15 (19)	95 (37)
Peritoneal dialysis	1 (5)	7 (9)	39 (15)
Kidney transplant	14 (64)	28 (36)	78 (30)
Not reported	1 (5)	21 (27)	2 (1)
Stage of CKD (patients with reduced kidney function only)			
Stage 1 (eGFR > 90 ml/min per 1.73 m^2^)	1 (25)	1 (1)	5 (10)
Stage 2 (eGFR 60–89 ml/min per 1.73 m^2^)	0 (0)	1 (1)	14 (29)
Stage 3 (eGFR 30–59 ml/min per 1.73 m^2^)	1 (25)	3 (4)	21 (44)
Stage 4 (eGFR 15–29 ml/min per 1.73 m^2^)	2 (50)	1 (1)	6 (12)
Stage 5 (eGFR< 15 ml/min per 1.73 m^2^ without dialysis or transplant)	0 (0)	0 (0)	2 (4)
Ethnicity^a^			
African, Afro-Caribbean	0 (0)	4 (4)	8 (2)
Arabic	0 (0)	2 (2)	2 (1)
Asian	0 (0)	4 (4)	45 (10)
Caucasian	22 (81)	55 (55)	301 (69)
Indigenous	2 (7)	8 (8)	73 (17)
Latin American	0 (0)	5 (5)	8 (2)
Pasifika	2 (7)	3 (3)	7 (2)
Not reported	2 (7)	25 (25)	1 (1)
Relationship with patient (caregiver only)			
Child	1 (25)	1 (4)	24 (14)
Friend	0 (0)	0 (0)	2 (1)
Parent	1 (25)	6 (26)	72 (41)
Spouse/partner	2 (50)	12 (52)	75 (43)
Relative	0 (0)	0 (0)	2 (1)
Not reported	1 (25)	4 (17)	1 (1)

CKD, chronic kidney disease; eGFR, estimated glomerular filtration rate.

Unknown or missing values are shown where participants did not respond to a particular item. Percentages may not sum to 100% because of rounding or multiple responses.

a Ethnicity was self-reported, and participants could select more than one option.

**Table 2:** Characteristics of health professionals

Characteristic	Round 1, *n* (%) 75 participants	Round 2, *n* (%) 53 participants	Round 3, *n* (%) 174 participants
Primary role			
Allied health	18 (24)	11 (21)	26 (15)
General physician	1 (1)	1 (2)	11 (6)
Nephrologist	16 (21)	10 (19)	47 (27)
Nurse	29 (39)	13 (25)	42 (24)
Researcher / Policy maker	11 (38)	11 (21)	40 (23)
Surgeon	0	0	7 (5)
Not reported	0	7 (13)	1 (1)
Age group (yrs)			
18–29	3 (4)	1 (2)	21 (12)
30–39	18 (24)	12 (23)	78 (45)
40–49	17 (24)	16 (31)	54 (31)
50–59	28 (36)	9 (19)	14 (8)
≥ 60	9 (12)	5 (9)	7 (5)
Not reported	0	10 (19)	0
Years of nephrology experience (clinicians only)			
≤ 10	20 (30)	10 (23)	57 (43)
11–20	20 (30)	14 (33)	49 (36)
21–30	19 (28)	7 (16)	20 (14)
≥ 30	9 (13)	6 (14)	8 (6)
Not reported	0	6 (14)	1 (1)

Unknown or missing values are shown where participants did not respond to a particular item. Percentages may not sum to 100% because of rounding or multiple responses.

### Nondialysis CKD

**Figure 3:**
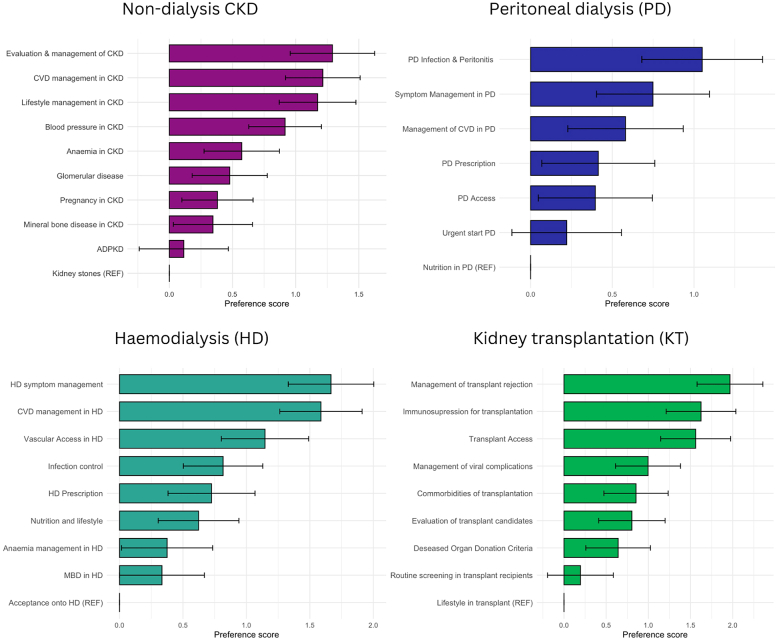
Round 1 Best-Worse Scaling (BWS) priorities by modules. *∗*Preference scores represent relative topic importance, error bars show within-group variability (points ± 95% confidence interval from multinomial logit), and the reference (REF) value indicates the lowest ranked topic, REF attribute fixed at 0; all scores are relative. Modules: Nondialysis CKD (purple), peritoneal dialysis (blue), hemodialysis (teal), kidney transplant (green). ADPKD, autosomal dominant polycystic kidney disease; CKD, chronic kidney disease; CVD, cardiovascular disease; MBD, mineral bone disease.

In round 1 (*n* = 102) 5 high priority broad topics were identified (Figure 3; Supplementary Table S1). The topics with the highest ranked preference score from BWS analysis were as follows (shown with their mean Likert scale rating ± SD and % agreement): (i) Evaluation and management of CKD (8.09 ± 1.11; 93%), (ii) Cardiovascular disease management in CKD (7.99 ± 1.14; 92%), (iii) Blood pressure management in CKD (7.93 ± 1.27; 88%), (iv) Nutrition and lifestyle in CKD (7.84 ± 1.35; 82%), and (v) Anemia management (7.5 ± 1.59; 75%). There were no significant differences between consumer and health professional Likert ratings (Supplementary Figure S1). For the BWS analysis, consumers ranked topics higher than health professionals except for the topic Cardiovascular disease management in CKD (Figure 1; Supplementary Figure S2).

In round 2 (*n* = 128) the broad priorities were refined into 31 subtopics (Supplementary Table S3). Subtopics meeting priority thresholds (mean ≥ 7.0; agreement ≥ 65%) included the following: (i) Treatment to prevent progression and complications (7.51 ± 2.00; 73%), (ii) Education and self-management (7.31 ± 1.88; 70%), (iii) Defining and evaluating CKD (7.16 ± 1.91; 70%), (iv) Medication management of blood pressure (7.16 ± 1.72; 75%), (v) Dietary patterns for CKD (7.25 ± 1.58; 74%), (vi) Lifestyle and exercise (7.01 ± 1.59; 70%), (vii) Lowering BP through lifestyle changes (7.08 ± 1.74; 66%), and (viii) Diagnosis and evaluation of anemia (7.09 ± 1.82; 66%).

**Figure 4:**
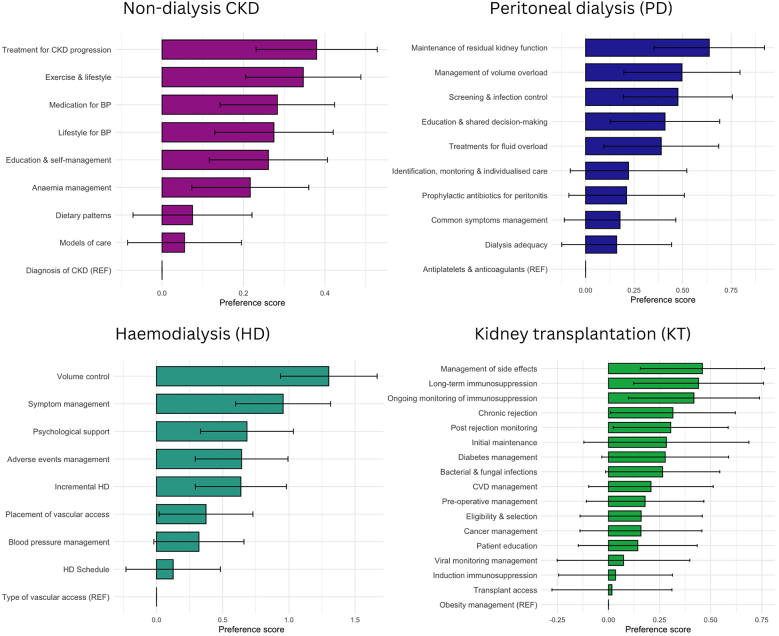
Round 3. Best-Worse Scales (BWS) across modules. ∗Preference scores represent relative topic importance (BWS, error bars show within-group variability (points ± 95% confidence interval from multinomial logit), and the reference (REF) value indicates the lowest ranked topic, REF attribute fixed at 0; all scores are relative. Modules: Nondialysis CKD (purple), peritoneal dialysis (blue), hemodialysis (teal), kidney transplant (green). CKD, chronic kidney disease; CVD, cardiovascular disease; BP, blood pressure.

**Figure 5:**
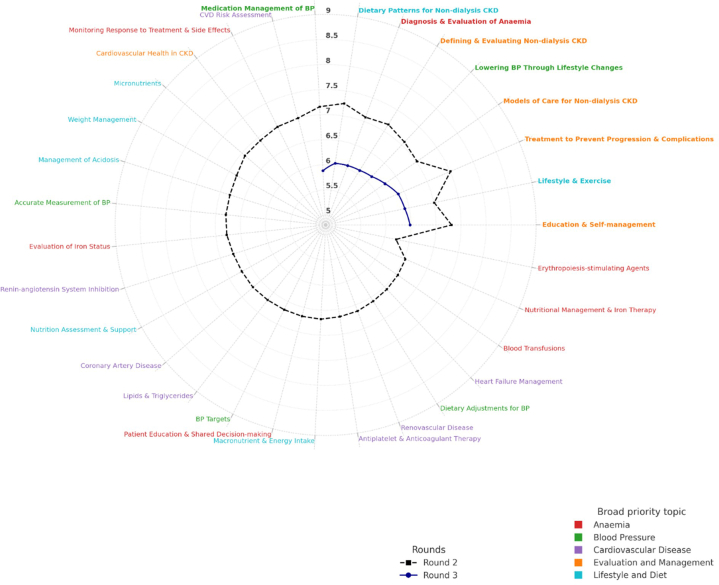
Mean Likert scores (9-point scale) for nondialysis CKD subtopics in rounds 2–3 (scale shown 5–9). R2 dashed; R3 solid. Sub-topics ordered by round 3 mean (descending) and then remaining round 2 mean (descending). Sample sizes, SD, and agreement^7^, ^8^, ^9^ in Supplementary Tables S3 and S7. Colors denote the round 1 broad priority topic area (see broad priority topic legend). BP, blood pressure; CKD, chronic kidney disease; PD, peritoneal dialysis.

By round 3 (*n* = 438) BWS preference scores demonstrated the following highest ranked topics: (i) Treatment strategies to prevent CKD progression’ (6.37 ± 2.53; 56%), (ii) Exercise and lifestyle management (6.41 ± 2.43; 57%), (iii) Medication for blood pressure (5.88 ± 2.67; 50%), (iv) Lifestyle management of blood pressure (6.13 ± 2.51; 51%), and (v) Education and self-management (6.48 ± 2.46; 59%) (Figure 4, Supplementary Table S4). Mean Likert scores were slightly lower than round 2, but ranking order was similar (Figure 5).

For the final 2 rounds, 96 free-text comments provided rationale and context. For the highest-rated subtopic, Treatment strategies to prevent CKD progression, participants described the impact of delayed intervention—“CKD has a drastic effect on a person’s life; any treatment to prevent progression is essential”—and stressed the need for early, coordinated care—“My husband’s CKD was caused by high blood pressure – he should have been on medications much earlier.” Other comments highlighted barriers such as, fatigue, low motivation, and limited support, and emphasized culturally appropriate, family-based education “Some people cannot self-manage – instead, there needs to be thinking around whānau [family] management.” Several participants reflected on the importance of communication “This is paramount … I have experienced not being heard and it was detrimental to my health” and called for realistic lifestyle support that includes sleep and pain management, in addition to weight-loss.

### PD

In round 1 (*n* = 84), all PD topics had mean Likert scores in the critically important range (≥ 7), with no differences between consumer and health-professional ratings (Supplementary Figure S1). The BWS preference scores and Likert ratings identified the following highest priority topics: (i) Management of infection and peritonitis (7.98 ± 1.41; 88%), (ii) Management of symptoms in PD (7.74 ± 1.31; 86%), (iii) PD prescription (7.70 ± 1.18; 82%), (iv) Access to PD (7.76 ± 1.20; 86%), and (v) Cardiovascular disease management (7.61 ± 1.29; 82%) (Figure 3, Supplementary Table S1).

**Figure 6:**
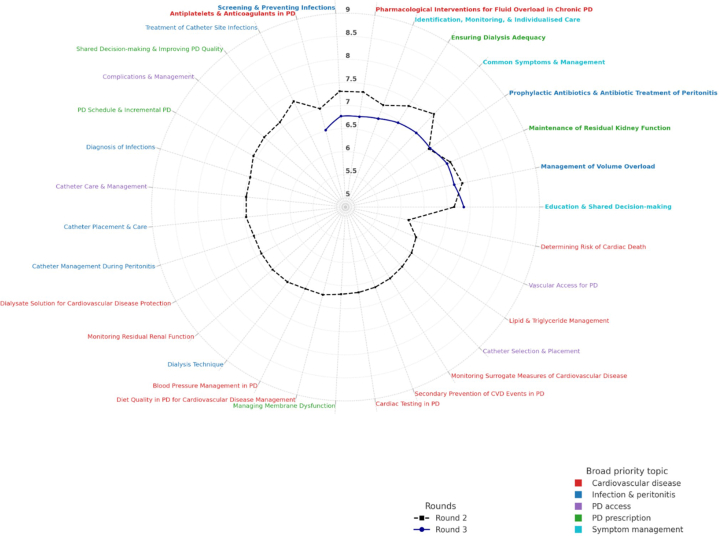
Mean Likert scores (9-point scale) for PD subtopics in rounds 2–3 (scale shown 5–9). R2 dashed; R3 solid. Sub-topics ordered by round 3 mean (descending) and then remaining round 2 mean (descending). Sample sizes, SD, and agreement^7^, ^8^, ^9^ in Supplementary Tables S4 and S7. Colors denote the Round 1 broad priority topic area (see broad priority topic legend). PD, peritoneal dialysis.

In round 2 (*n* = 48), priority topics were refined into 31 subtopics (Figure 6; Supplementary Table S5). The subtopics that met the criteria for high-priority were as follows: (i) Common symptoms and management (7.58 ± 1.31; 77%), (ii) Management of volume overload (7.38 ± 1.82; 75%), (iii) Ensuring dialysis adequacy (7.38 ± 1.69; 69%), (iv) Maintenance of residual kidney function (7.27 ± 1.61; 65%), (v) Identification, monitoring, and individualized care (7.15 ± 1.38; 73%), and (vi) Prophylactic antibiotics and antibiotic treatment of peritonitis (7.00 ± 1.82; 73%). Antiplatelets and anticoagulants in PD (7.00 ± 1.97; 58%) did not reach consensus but was retained for inclusion in round 3 because of higher consumer rating (8.38 ± 1.2; 68%).

Round 3 (*n* = 96) reaffirmed the previous priorities (Figure 6), mean Likert scores were slightly lower, but rankings remained similar. BWS preference scores identified these high priority topics as follows: (i) Maintenance of residual kidney function in PD (7.19 ± 2.10; 77%), (ii) Management of volume overload in PD (7.20 ± 1.84; 68%), (iii) Screening and infection control in PD (6.77 ± 2.29; 67%), (iv) Education and shared-decision making (7.37 ± 1.98; 74%), and (v) Pharmacological treatments for fluid overload (6.78 ± 2.33; 65%) (Figure 4; Supplementary Table S4). Consumers rated most topics higher than health professionals, except Maintenance of residual kidney function and Prophylactic antibiotics for peritonitis (Supplementary Figure S3).

Six PD comments focused on access and service delivery, highlighting long waits for PD catheter insertion in regional areas (“In the last two years it has taken anywhere from 11 days [private] to 188 days [public] from referral to insertion date”).

### HD

In round 1 (*n* = 101), all HD topics were rated as critically important (Likert ≥ 7). The top-rated and ranked topics (Likert and BWS) were as follows: (i) Cardiovascular disease management in HD (7.96 ± 1.33; 89%), (ii) HD prescription (7.85 ± 1.16; 92%), (iii) HD vascular access (7.84 ± 1.24; 85%), (iv) HD symptom management (7.83 ± 1.18; 88%), and (v) Acceptance onto HD (7.57 ± 1.37; 78%) (Figure 3; Supplementary Table S1).

**Figure 7:**
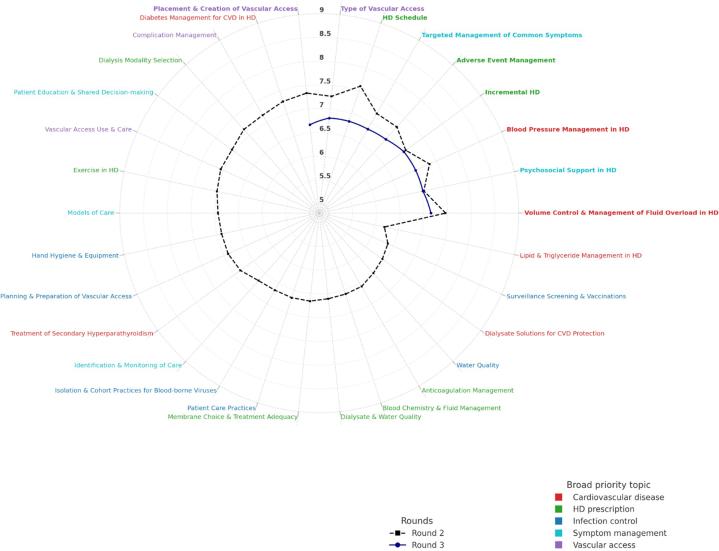
Mean Likert scores (9-point scale) for nondialysis HD subtopics in rounds 2–3 (scale shown 5–9). R2 dashed; R3 solid. Sub-topics ordered by round 3 mean (descending) then remaining round 2 mean (descending). Sample sizes, SD, and agreement^7^, ^8^, ^9^ in Supplementary Tables S5 and S7. Colors denote the round 1 broad priority topic area (see broad priority topic legend). HD, hemodialysis

In round 2 (*n* = 68), 30 subtopics were evaluated (Figure 7; Supplementary Table S6). Highest-rated subtopics were as follows: (i) Dialysis schedule (7.61 ± 1.59; 73%), (ii) Volume control and management of fluid overload in HD (7.46 ± 1.96; 78%), (iii) Blood pressure management in HD (7.34 ± 1.51; 76%), (iv) Placement and creation of vascular access (7.34 ± 1.65; 70%), (v) Type of vascular access (7.27 ± 1.81; 66%), (vi) Symptom management in HD (7.22 ± 1.87; 68%), (vii) Psychological support in HD (7.05 ± 1.78; 66%), (viii) Adverse event management (7.24 ± 1.57; 66%), and (ix) Incremental HD (7.05 ± 1.85; 66%).

Round 3 (*n* = 167) reaffirmed most of the round 2 priority order (except “Dialysis schedule”; it did not meet round 3 BWS threshold), but with lower mean Likert scores (Figure 7). The top BWS-ranked subtopics were as follows: (i) Volume control and management of fluid overload in HD (7.15 ± 2.12; 75%), (ii) Symptom management in HD (6.84 ± 2.46; 64%), (iii) Psychological care in HD (7.03 ± 2.30; 69%), (iv) Adverse event management (6.89 ± 2.47; 72%), and (v) Incremental HD (7.00 ± 2.28; 69%) (Figure 4; Supplementary Table S4).

Nine free-text comments were submitted, reinforcing the importance of symptom management (“So important for improving the patient’s life”), restless leg syndrome and poor sleep, and calling for “more individualized fluid targets and management” and “toxin removal for better cardiovascular diseases outcomes.”

### Kidney Transplantation

In round 1 (*n* = 100), all topics were rated critically important (≥ 7). Top-ranked were “Transplant rejection” (8.36 ± 1.05; 96%), “Immunosuppression” (8.21 ± 1.18; 94%), “Access to transplantation” (7.66 ± 1.46; 82%), “Viral complications” (7.93 ± 1.09; 87%), and “Comorbidity management” (8.08 ± 1.08; 89%) (Figure 3; Supplementary Table S1). In round 1, this was the only module in which some mean Likert scores differed between consumers and health professionals (Supplementary Figure S1).

**Figure 8:**
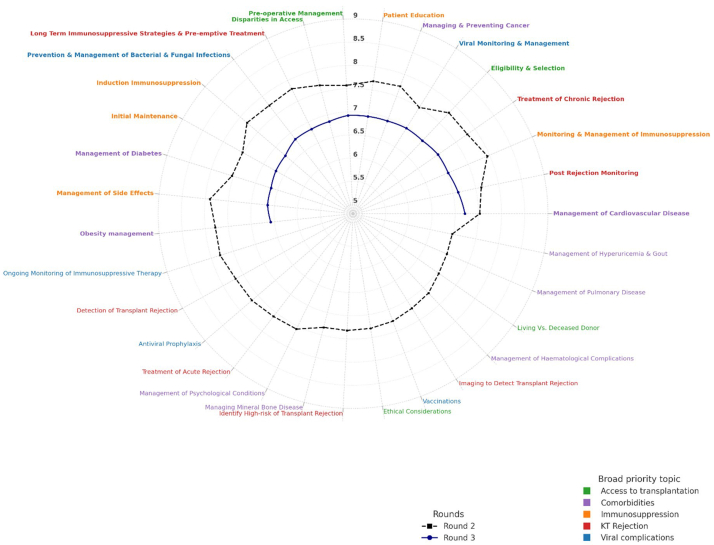
Mean Likert scores (9-point scale) for Transplant subtopics in rounds 2–3 (scale shown 5–9). R2 dashed; R3 solid. Sub-topics ordered by round 3 mean (descending) then remaining round 2 mean (descending). Sample sizes, SD, and agreement^7^, ^8^, ^9^ in Supplementary Tables S6, S7. Colors denote the round 1 broad priority topic area (see Broad priority topic legend). round 3 “Ongoing management of immunosuppression” consolidated two round 2 subtopics, “Monitoring & management of immunosuppression” (anchor) and “Ongoing monitoring of immunosuppressive therapy”. The round 3 mean is shown once at the anchor node; both round 2 items remain for context.

Round 2 (*n* = 71) assessed 31 subtopics (Figure 8; Supplementary Table S7). Seventeen met high-priority thresholds (mean ≥ 7.5; agreement ≥ 75%), including Monitoring and management of immunosuppression (7.95 ± 1.15; 88%), Management of medication side-effects (7.90 ± 1.32; 88%), Eligibility and selection for transplant (7.80 ± 1.16; 88%), Ongoing monitoring of immunosuppressive therapy (7.80 ± 1.25; 84%), Treatment of chronic rejection (7.80 ± 1.38; 82%), Management of obesity (7.78 ± 1.17; 84%), Prevention and management of bacterial and fungal infections (7.75 ± 1.19; 84%), Managing and preventing cancer (7.73 ± 1.47; 84%), and Detection of transplant rejection (7.69 ± 1.60; 78%). As per the methods, two overlapping round 2 subtopics were merged into “Ongoing management of immunosuppression” for round 3 to avoid duplication in the BWS task. In both Rounds 1 and 2, transplant had the highest mean scores compared with the other modules.

By round 3 (*n* = 162) (Figure 8), the priority subtopics were Management of medication side-effects (6.65 ± 2.53; 65%), Strategies for long-term immunosuppression (6.83 ± 2.27; 63%), Ongoing monitoring of immunosuppression (7.03 ± 2.04; 66%), Treatment of chronic rejection (7.03 ± 2.37; 69%), and Post-rejection monitoring (7.11 ± 2.17; 72%) (Figure 4). Likert scores were slightly lower than round 2 (Figure 8), and the wider CIs indicated variability in preferences, particularly among consumers versus health-professionals. Consumers prioritized Management of side-effects, Cancer prevention, and Infection control, whereas clinicians emphasized Eligibility and selection for transplantation (Supplementary Figures S3 and S4).

Twenty free-text comments (rounds 1 and 2) were centered on side-effects, immunosuppression, and rejection. Participants described side-effects as horrendous and emphasized better education and troubleshooting support managing difficult symptoms. One caregiver reflected—“The increase in immunosuppressant medication has caused extreme skin cancer on my husband’s face, requiring multiple operations.” Others highlighted unmet evidence needs, including viral monitoring and BK virus management.

### Additional Topics

Overall, 627 additional topics with Likert ratings were suggested (Round 1 = 80, Round 2 = 30, Round 3 = 517) most reiterating existing priorities. In the final round, a small number of new areas were proposed, psychosocial care, care coordination, oral health, shared decision-making, equity for specific populations. Several respondents also highlighted the need for family-orientated approaches for the Māori and Aboriginal and Torres Strait Islander communities (“Some people cannot self-manage – instead, there needs to be thinking around whānau [family] management”). No new topics were raised by ≥ 10% of participants.

## Discussion

This study identified high-priority topics for nephrology guidance using a 3-round modified Delphi (9-point Likert ratings and BWS), integrating consumers and health professionals’ perspectives across nondialysis CKD, PD, HD, and kidney transplantation. Priorities largely focused on slowing CKD progression and improving symptoms while receiving kidney replacement therapy; aligning with shifts towards patient-centered care.^12^^,^^14^^,^^21^^,^^28^

The emphasis on the topics Treatment to slow CKD progression, Maintenance of residual kidney function in PD, Volume control in HD, and Side-effects management in transplantation reflects both clinical priorities and lived experience. Consumers emphasized psychosocial support and shared decision-making, reflecting unmet needs that warrant greater attention in guideline development and implementation strategies. These priorities echo findings from other kidney research prioritization efforts, including Standardized Outcomes in Nephrology, Australian musculoskeletal guidelines, and Canadian Society of Nephrology Guideline initiatives, highlighting consistent emphasis on symptoms, side-effects, and treatment burden.^15^^,^^21^^,^^22^^,^^28^, ^29^

A limitation of this study is the potential selection bias inherent to online voluntary participation. Despite broad recruitment and consumer incentives, those with strong interest or specific experiences may have been more inclined to participate. The predominance of Australia respondents and smaller representation from New Zealand may limit the generalizability of some priorities to other healthcare systems and cultural contexts.

Before the Delphi rounds, the initial list of broad topics was derived from existing guidelines, and there is a possibility that some topics may have been misclassified or omitted. Although participants were able to suggest additional topics, some perspectives, particularly consumers, may have been under-represented during early topic selection because of perceived lack of knowledge or expertise, alongside the higher proportion of health professionals in round 1. Additionally, aspects of kidney supportive care and conservative kidney management were embedded within broader domains, these were not presented as standalone topics. Framing them independently may have yielded different prioritization patterns; however, few participants nominated supportive or conservative care as new topics in later rounds, suggesting these needs were largely captured within existing categories.

The exclusive use of online surveys may also have excluded people with limited digital access or literacy, particularly in rural, remote, or socioeconomically disadvantaged settings. Snowball sampling, although used to increase recruitment, may have introduced some homogeneity of perspectives, as participants recruited through professional or social networks may share similar views. Although the responses were deidentified, there remains potential for authority or conformity bias, whereby participants, particularly clinicians, may align their responses with perceived prevailing opinions within their networks. However, consumer participation increased across rounds, with a higher proportion of caregivers contributing unique perspectives. In round 1, there was good alignment between consumers and health professionals in topic ratings and rankings. Greater heterogeneity between groups was observed in round 3, particularly for transplant subtopics, as reflected by wider confidence intervals. This likely reflects genuine variation in preferences because of broader sampling, with priorities differing by participant role (donor, patient, caregiver, and health professional).^30^, ^31^, ^32^

Engagement strategies should be tailored to better reach marginalized populations to ensure their perspectives are adequately incorporated in priority-setting exercises, and that culturally safe approaches are used when working with Aboriginal and Torres Strait Islander and Māori communities, who are disproportionately affected by CKD. Ensuring that updated, evidence-based guidance reflects their priorities may support more equitable care and improved patient outcomes.^33^ Notably, participation by self-identified indigenous people in round 3 exceeded Australian population proportions, highlighting the importance of inclusive recruitment and the relevance of CARI’s approach to developing CKD-specific guidelines in partnership with indigenous communities and aligned with their priorities.^33^, ^34^, ^35^

A further challenge in CKD guideline development is the breadth of topics and variable pace of evidence generation. Some areas have rapid trial output, whereas others draw from a relatively unchanging evidence-base. A unique strength of our study was the foundational evidence mapping, which distilled broad guideline topics into refined subtopics aligned with specific clinical questions. Emerging “Living” guideline and systematic review methods rely on clearly defined, up-to-date priorities to remain relevant and impactful.^36^, ^37^, ^38^, ^39^ The priorities identified in this survey provide a clear pathway to focus on high-impact topics, improving the relevance, transparency, and uptake of guideline recommendations.^5^^,^^15^^,^^16^ There is a growing shift towards rapid, focused updates of high-priority guideline topics, as recently employed by Kidney Disease: Improving Global Outcomes in their glomerular diseases guideline,^40^^,^^41^ which ensures clinical guidance remains current and responsive to new emerging evidence.^38^^,^^42^^,^^43^ This model, successfully implemented during the COVID-19 pandemic and piloted in nephrology, offers a promising approach to support best care for people with CKD.^43^, ^44^, ^45^

The integration of BWS alongside Likert ratings improved discrimination between closely ranked items and clarified differences between consumer and health-professional preferences. Free-text comments provided essential explanatory context and helped in surfacing equity-related needs not captured in quantitative scores alone. For example, within Education and self-management, several comments highlighted whānau-centered (family-centered) education for indigenous (Māori) communities,^33^ an equity consideration not apparent from ratings alone. The large number of additional topic suggestions also reflects strong participant engagement. These suggestions largely affirmed the shortlisted subtopics, strengthening the final prioritized list.

Through this rigorous prioritization process, we identified high-priority subtopics across the spectrum of CKD that should be addressed by systematic reviews and guidelines. These priorities emphasize slowing CKD progression and improving symptoms and side-effects in kidney replacement therapy. By iteratively narrowing broad domains into specific, actionable subtopics, this study provides a framework for informing future guideline prioritization processes. The prioritized subtopics offer a clear foundation for targeted updates that will inform future research and policy, aiming to improve care and outcomes for people living with CKD.

## Appendix

### Members of the CARI Guidelines Steering Committee

Bhadran Bose, Leanne Brown, Helen Coolican, Vanessa Cullen, Jeffrey Ha, Kelly Lambert, Casey Light, Min Jun, Thu Nguyen, Emmy O’Neill, Emily See, Carla Scuderi, and Andrea Viecelli.

## Disclosure

All the authors declared no competing interests.

## Supplementary Material

Supplementary File (PDF) **File S1.** Protocol. **Figure S1.** Round 1: Mean Likert scale difference- consumer and health professionals. **Figure S2.** Round 1: BWS preference scores by groups- consumer and health professionals. **Figure S3.** Round 3: Mean Likert scale difference- consumer and health professionals. **Figure S4.** Round 3: BWS by module - consumer vs health professionals. **Table S1.** Accurate Consensus Reporting Document checklist. **Table S2.** Summary statistics for round 1 Likert scores. **Table S3.** Summary statistics for round 2 non-dialysis CKD Likert scores. **Table S4.** Summary statistics for round 2 PD Likert scores. **Table S5.** Summary statistics for round 2 HD Likert scores. **Table S6.** Summary statistics for round 2 Transplant Likert scores. **Table S7.** Summary statistics for round 3 guideline subtopics by module.

## Footnotes

Supplementary File (PDF)
**File S1.** Protocol. **Figure S1.** Round 1: Mean Likert scale difference- consumer and health professionals. **Figure S2.** Round 1: BWS preference scores by groups- consumer and health professionals. **Figure S3.** Round 3: Mean Likert scale difference- consumer and health professionals. **Figure S4.** Round 3: BWS by module - consumer vs health professionals. **Table S1.** Accurate Consensus Reporting Document checklist. **Table S2.** Summary statistics for round 1 Likert scores. **Table S3.** Summary statistics for round 2 non-dialysis CKD Likert scores. **Table S4.** Summary statistics for round 2 PD Likert scores. **Table S5.** Summary statistics for round 2 HD Likert scores. **Table S6.** Summary statistics for round 2 Transplant Likert scores **Table S7.** Summary statistics for round 3 guideline subtopics by module.
