# Supplementary material for: Priorities for Evidence-Based CKD Guidance Using Delphi Methods
Source: Kidney Int Rep. 2026 May 6;11(8):106573. doi: 10.1016/j.ekir.2026.106573 (PMC13310944; doi:10.1016/j.ekir.2026.106573)
Supplement: Supplementary File (PDF) — File S1. Protocol. Figure S1. Round 1: Mean Likert scale difference- consumer and health professionals. Figure S2. Round 1: BWS preference scores by groups- consumer and health professionals. Figure S3. Round 3: Mean Likert scale difference- consumer and health professionals. Figure S4. Round 3: BWS by module - consumer vs health professionals. Table S1. Accurate Consensus Reporting Document checklist. Table S2. Summary statistics for round 1 Likert scores. Table S3. Summary statistics for round 2 non-dialysis CKD Likert scores. Table S4. Summary statistics for round 2 PD Likert scores. Table S5. Summary statistics for round 2 HD Likert scores. Table S6. Summary statistics for round 2 Transplant Likert scores. Table S7. Summary statistics for round 3 guideline subtopics by module. [file mmc1.pdf]

Supplementary files:

S1 file: Protocol

Updated evidence informing clinical care priority setting for chronic kidney disease. Protocol

Introduction

The rapid proliferation of scientific literature has further complicated the translation of new evidence into clinical practice (50), estimated to take up to 17 years (51). Living evidence uses continuous evidence surveillance and rapid response pathways to incorporate new relevant data into evidence-based guidance for clinical care (40) to overcome these challenges without sacrificing methodological rigour. Given the limited resources available, a prioritisation process is required to maximise the support and impact of systematic and rigorously appraised resources relevant to the kidney health community.

Cochrane reviews are internationally recognised as the highest standard in evidence-based health care and should be relevant and up-to-date for healthcare and policy. However, topics are usually decided from 'bottom-up', by potential authors, based upon their personal interest and with little involvement from end-users of the reviews, including patients. Less than half of Cochrane review groups have a mechanism for selecting and prioritising topics for Cochrane reviews. Similarly, the Australian and New Zealander guideline developers in chronic kidney disease, CARI Guidelines have a top-down approach with the current process for selecting clinical practice guidelines is decided by the CARI Guidelines Steering Committee, made up of kidney specialists and nurses, consumers, and researchers. The decision is made on the community's need for guidance on proposed clinical practice guidelines. At present, there is no external stakeholder input for prioritising clinical practice guideline topics to be undertaken by CARI Guidelines.

CARI Guidelines and Cochrane Kidney and Transplant are committed to ensuring that our limited resources are directed towards high-impact synthesis evidence and recommendations for care for end-users. To respond to the kidney health community's priorities, CARI Guidelines and Cochrane Kidney and Transplant will undertake a prioritisation project.

Methods

We have adapted guidance from the Cochrane priority setting. The principles listed in Table 1, and the adapted 5-step process listed in Table 2 will be adhered to.

Table 1. Guidance notes standards for priority settings (1)

	Mandatory standards	Highly desirable standards
Governance	1. A team to lead the priority setting process. The Steering	2. Include external stakeholders including

	Mandatory standards	Highly desirable standards
	Committee subcommittee (including consumers) will help define and refine the scope of the exercise	consumers in the priority setting Steering Committee
Stakeholder engagement	3. Include stakeholder groups, for example, systematic review developers, people with lived experience of disease, clinicians etc.<br>4. Publish the intention to undertake priority setting through existing communication and dissemination channels (ANZSN, RSA, KHA) to engage with stakeholders to get involved	5. Engagement with multiple stakeholder groups e.g., external guideline developers, funders, consumer organisations, professional societies
Documentation and dissemination	6. Document the stakeholder engagement, methods and criteria for future priority setting.<br>7. Disseminate findings of the priority setting back to stakeholders and participants	8. Publish a detailed report of priority-setting exercise in a scientific journal.<br>9. Evaluate the priority setting process and outcomes
Currency and timeframe	10. Priority-setting should be repeated at regular intervals according to emerging treatment within the field of chronic kidney disease	11. Priority-setting should occur every five years

Table 2. Adapted guidance note 5-step process.

	Mandatory standards
**Step 1:** picking a scenario that best reflects your situation	1. Documenting an existing priority setting process to adhere to mandatory standards.<br>2. Quick update via a rapid review and prioritisation of existing clinical practice guidelines<br>3. Thorough update and revision of existing clinical practice guidelines<br>4. Quick development of new clinical practice guideline topics<br>5. Thorough development of new clinical practice guideline topics
**Step 2:** Scoping a priority setting process	• Purpose<br>• Intersection with existing review titles and clinical practice guidelines<br>• Governance, team, funding, timeframe<br>• Stakeholders<br>• Geographic and thematic scope

	Mandatory standards
**Step 3:** Developing a plan for the priority setting process and deciding of methods for prioritisation	• Collecting all systematic reviews topics and clinical practice topics<br>• Cleaning the data<br>• Prioritisation of topics<br>• Devising answerable and feasible clinical practice guideline topics<br>• Implementation
**Step 4:** Documenting the priority setting process and sharing the results with relevant stakeholders	• Mandatory implementation standards listed in Table 1<br>• Highly desirable implementation standards listed in Table 1
**Step 5:** Evaluating the process, monitoring implementation, and deciding when the prioritised need to be revisited	• Monitoring the implementation of the prioritised reviews<br>• Monitoring and evaluation of outcomes<br>• Monitoring the research field

Step 1: Picking a scenario that best reflects your situation.

The scenario for prioritisation process is best reflected in the scenarios listed below.

- Scenario 3: Thorough update and revision of existing clinical practice guidelines and systematic reviews
- Scenario 5: Thorough development of new clinical practice guideline and systematic review topics

Step 2: Scoping a priority setting process.

Purpose

Objective: to systematically develop and implement a priority setting process that identifies important topics required for updated evidence synthesis for the domains of chronic kidney disease, dialysis, kidney transplantation, to ensure effective distribution of limited CARI Guideline resources.

Aims:

- Engage and involve stakeholders of Cochrane Kidney and Transplant and CARI Guidelines in priority setting, particularly for consumers.
- Generate a list of high-priority clinical questions across all domains for developing updated evidence-synthesised outputs, such as systematic reviews and clinical practice guidelines.
- Evaluate the prioritisation process and improve implementation to ensure future streamlined prioritisation processes for clinical questions for kidney disease.

Intersection with existing systematic reviews and clinical practice guidelines

We will provide a list of Cochrane Kidney and Transplant systematic reviews, and clinical practice guideline topics in kidney disease.

Governance

Based on the mandatory standards, we will establish a team to lead the prioritisation process, involving Cochrane Kidney and Transplant and members of the CARI Guidelines Steering Committee and staff. The members should include all relevant stakeholders and include geographical representation across Australia and New Zealand.

Team

The **Steering Committee** will help provide oversight and contribute to the inception, planning and execution of the prioritisation project. David Tunnicliffe will oversee the management of the project in his role as Scientific Director at CARI Guidelines. Brydee Cashmore will undertake the project as part of her PhD candidature. Martin Howell will provide expertise in prioritisation-setting methods. There will be both consumer and clinician (persons TBC) input to help develop and interpret the analysis of the surveys.

Stakeholders

Stakeholders from the CARI Guidelines Steering Committee, and Cochrane Kidney and Transplant management board will form the Steering Committee for the project. The Steering Committee represents stakeholders from the Australian New Zealand Society of Nephrology, Renal Society of Australasia, and people with lived experience of kidney disease. Other external stakeholder organisations, such as Kidney Health Australia, and other professional societies will be engaged in the dissemination phases of the project.

Geographic and thematic scope

Cochrane Kidney and Transplant Editorial Board is represented by 3 WHO regions, CARI Guidelines Steering Committee, represents Australian and New Zealand stakeholders. All stakeholders will be engaged and provided the opportunity to review and provide feedback on the protocol and involved in the consensus agreement process of the results. The findings of the prioritisation exercise will be shared among professional societies and key consumer organisations in the region.

Funding

Internal sources of support include.

- NHMRC Emerging Leadership 1 Investigator Grant (APP1197337)
- Australian Living Evidence Consortium (ALEC) – Developing Australian Living Kidney Guidelines
- NHMRC [GO3093] for salaries contributing to the CKT Managing Editor
- NSAP [4-E85UZWV] for salaries contributing to the CKT Copy Editor, Information Specialist, Senior Consumer Specialist

In-kind sources of support:

- All Steering Committee members

<u>Step 3: Developing a plan for priority setting process and deciding on methods for prioritisation.</u>

Collating clinical practice guidelines and systematic reviews

- We will list all current chronic kidney disease guidelines and existing Cochrane protocols, reviews, and updates.
- We will compile existing data on the access, citations and impact in policy documents on all Cochrane Kidney and Transplant systematic reviews and CARI Guidelines.

- We will use our in-house Register of Kidney trials to integrate new and upcoming trials into our priority-setting process directly. We will extract a list of ongoing and expected trials in each topic area to inform which reviews would require updating and highlighting when there is no systematic review, but sufficient trials are available.
- We will compare our methods and processes to that of The SONG Initiative(52). We will examine how priorities were set, what methods were used, and whether stakeholder perspectives were adequately included.

Developing the list of initial topics for round one Delphi survey

- We will develop a broad list of around 20 topics for each module peritoneal dialysis (PD) (pilot survey), haemodialysis (HD), transplant, and chronic kidney disease (CKD) used in round one of the surveys. The list will be developed from the described mapping process of current guideline topics and existing Cochrane systematic reviews.
- The final list will be reviewed by a clinical and consumer input.

Prioritisation and refining the topics and healthcare questions.

A list of topics for evidence synthesis will be identified with a multi-round Delphi online survey. The Steering Committee will then review and refine the list, removing duplicates and irrelevant questions through group consensus. We will clearly document the process of topic and question removal for transparency. Reasons for removal include:

- High-quality systematic reviews and clinical practice guidelines already exist, and new trials are unlikely.
- Topics are beyond the scope of the guidelines.
- Topics are unclear or ill defined.
- Duplications of topics and questions collected.

Once the list of agreed topics and clinical questions for evidence synthesis are finalised, for each of the domains (CKD, HD, PD, kidney transplantation), we will apply the Delphi method to distribute an online survey.

Methods for the Delphi Survey

A three-round online Delphi survey will be conducted to gain transparent consensus and maximise participation of all stakeholders. The Delphi survey will involve three rounds of sequential surveys (Figure 1).

Inclusion criteria: English speaking adults with lived experience of CKD (across the full spectrum) or caregivers (18 years of age or older) and health professionals (physicians, surgeons), nurses, allied health professionals, policy makers/regulators involved in the management of chronic kidney disease are eligible to participate.

Recruitment: We will recruit health professionals and adults with lived experience of CKD internationally through the Australian and New Zealand Society of Nephrology (ANZSN), Renal Society of Australasia (RSA), Kidney Health Australia (KHA), SONG database, CARI Guidelines and Cochrane Kidney Transplant databases. Standard invitations will be used across these mailing list, inviting people to complete the Delphi survey hosted online by Qualtrics.

We aim to recruit approximately 120 adults (aged 18 years and over) with lived experience of chronic kidney disease (both patients and their caregivers). A general invitation will be circulated via email/post to their membership database of patients and caregivers, inviting them to participate in the surveys. Patients and caregivers will be invited to contact ANZSN, RSA, KHA, etc. if they are interested in participating in the surveys. Subsequently, they will be provided with the details of the researchers and invite patients/caregivers to contact the investigators directly to discuss the study.

Additionally, approximately 120 health professionals and consumers involved in the development of evidence synthesis of chronic kidney disease (nephrologists, nurses, surgeons, and policymakers) will be invited to participate. Potential participants will be identified via the investigators existing professional networks. The investigators will approach potential participants via email, or telephone to determine if they are interested in participating the Delphi surveys hosted online via Qualtrics.

Sample size: There is no standard sample size calculation in Delphi processes for prioritising clinical guideline topics though Delphi studies in core outcomes development have reported numbers ranging from 13 to 222. We will aim to recruit 300 Delphi respondents with patients/caregivers comprising at least one third of the total sample size to ensure a numerical balance between patients/caregivers and health professionals. Thus, we will aim to invite patients, caregivers, clinicians (nephrologists, vascular surgeons, nurses, psychologists, social workers, and dieticians, researchers, and policy makers), to broadly reflect the workforce proportions.

Ethics: Ethics has been sought from The University of Sydney in 2023. Participants will be provided with a participant information and consent form in the survey. Participants will respond to a consent statement at the commencement of the Delphi survey.

Delphi rounds

Figure 1. Overview of Delphi Process

<u>Round 1:</u> This will cover: CKD, Dialysis (including PD and HD modules) and Kidney transplantation. Participants will rate the importance of each "updating of evidence" topic using the GRADE recommended 9-point Likert scale. Rankings of 7-9 will indicate topics of critical importance, 4-6 are

important but not critical, 1-3 rankings as of limited importance (topics identified by previous priority setting exercises). There will be approximately 10-15 broad topics devised from the mapping process for each module. To minimise the risk of ordering bias, the topics within the domains will be randomised. For each topic, a text box is provided for participants to provide comments about their choices. After the rating scales are completed, participants can provide justification of ranking and suggest additional guideline and systematic review topics. All research questions or topics that are suggested by more than 10% of the participants, and do not duplicate topics in the original survey, will be included and carried through to round 2 of the Delphie survey.

Criteria for inclusion and exclusion in round 2 will be determined based on the distribution of the data. An example of selection criteria from the SONG Outcome Delphi is described below.

- Consensus in: 70% participants scoring as 7 to 9 AND 15% participants scoring 1 to 3.
- Consensus out: 70% participants scoring 1 to 3 AND 15% participants scoring 7 to 9.
- If more than >10% of participants suggested a new clinical practice guideline topic, it will be included in round 2.

<u>Round 2:</u> For Round 2, the broad topics identified as priorities in Round 1 will be mapped to specific research questions related to existing systematic reviews and clinical practice guidelines. Participants will be asked to rank the importance of the research questions, with separate consumer and clinician groups. De-identified comments provided for rating justifications in round 2 will also be presented in a table, as well as the new suggested topics (see criteria listed above). Best worse scales will also be included to further identified top rated preferences for topics on clinical practice guidelines.

<u>Round 3:</u> A list of top ranked score for the topics for each module (CKD, dialysis, and transplant) from round 2 will be presented. Participants will review the scores and re-rank the topics using the 9-point Likert scale, they can also explain their reasons for changing their scoring and will be available in round 3. Best worse scales will also be included in Round 3 to further identified top rated preferences for topics on clinical practice guidelines. Participants will be re ranked using 9-point Likert scale and best worse scale used.

Each round will be 3 weeks in duration, one month apart.

Devising answerable and feasible topics for evidence synthesis

The CARI Guidelines Steering Committee and Cochrane Kidney and Transplant will review the appropriateness and feasibility of the proposed topic for updated evidence-based synthesis and guidance separately.

Step 4: Documenting the priority setting process and sharing the results with relevant stakeholders.

The results of the prioritisation survey will be shared with stakeholders through appropriate newsletters, emails, and reports. We will publish a detailed report on the priority setting exercise on our website and a summary report in relevant peer-reviewed journals. We will notify stakeholders when these are published an available for access. We will develop a plan for how the priority topics will be delivered.

Step 5: Evaluating the process, monitoring implementation, and deciding when priorities need to be revised.

To inform future prioritisation exercises we will invite participants of the Delphi surveys and relevant stakeholders to complete feedback via an online survey. Additionally, we will examine whether the standards (both mandatory and highly desirable) were met following the completion of the exercise.

We will also monitor:

- The delivery of prioritised topics for clinical practice guidelines and systematic reviews
- Document the dissemination and implementation of prioritised clinical practice guidelines – journal publications, presentations, social media activity, citations.

Revising the prioritisation process

The adapted Cochrane Guidance indicates that the mandatory standard of prioritisation setting exercise should be undertaken every five years, with every three years being highlight desirable. CARI Guidelines Steering Committee and Cochrane Kidney and Transplant will decide when an updated prioritisation process should be undertaken and what methods should be used separately.

Table S1: Accord reporting checklist

Item No.		Section	Checklist Item (*help text*)	Page No.
T1		**Title**	Identify the article as reporting a consensus exercise and state the consensus methods used in the title. *For example, Delphi or nominal group technique.*	1
I1		**Introduction**	Explain why a consensus exercise was chosen over other approaches.	5
I2			State the aim of the consensus exercise, including its intended audience and geographical scope (national, regional, global).	5-6
I3			If the consensus exercise is an update of an existing document, state why an update is needed, and provide the citation for the original document.	n/a
M1		**Methods** Registration	If the study or study protocol was prospectively registered, state the registration platform and provide a link. If the exercise was not registered, this should be stated. *Recommended to include the date of registration.*	8
M2		Selection of SC and/or panellists	Describe the role(s) and areas of expertise or experience of those directing the consensus exercise. *For example, whether the project was led by a chair, co-chairs or a steering committee, and, if so, how they were chosen. List their names if appropriate, and whether there were any subgroups for individual steps in the process.*	7-8
M3			Explain the criteria for panellist inclusion and the rationale for panellist numbers. State who was responsible for panellist selection.	7
M4			Describe the recruitment process (how panellists were invited to participate). *Include communication/advertisement method(s) and locations, numbers of invitations sent, and whether there was centralised oversight of invitations or if panellists were asked/allowed to suggest other members of the panel.*	7
M5			Describe the role of any members of the public, patients or carers in the different steps of the study.	7
M6		Preparatory research	Describe how information was obtained prior to generating items or other materials used during the consensus exercise. *This might include a literature review, interviews, surveys, or another process.*	6
M7			Describe any systematic literature search in detail, including the search strategy and dates of search or the citation if published already. *Provide the details suggested by the reporting guideline PRISMA and the related PRISMA-Search extension.*	6,8
M8			Describe how any existing scientific evidence was summarised and if this evidence was provided to the panellists.	8
M9		Assessing consensus	Describe the methods used and steps taken to gather panellist input and reach consensus (for example, Delphi, RAND-UCLA, nominal group technique). *If modifications were made to the method in its original form, provide a detailed explanation of how the method was adjusted and why this was necessary for the purpose of your consensus-based study.*	9
M10			Describe how each question or statement was presented and the response options. State whether panellists were able to or required to explain their responses, and whether they could propose new items.	7 and 9

Item No.		Section	Checklist Item (*help text*)	Page No.
			Where possible, present the questionnaire or list of statements as supplementary material.	
M11			State the objective of each consensus step. *A step could be a consensus meeting, a discussion or interview session, or a Delphi round.*	6
M12			State the definition of consensus (for example, number, percentage, or categorical rating, such as 'agree' or 'strongly agree') and explain the rationale for that definition.	9
M13			State whether items that met the prespecified definition of consensus were included in any subsequent voting rounds.	9
M14			For each step, describe how responses were collected, and whether responses were collected in a group setting or individually.	7-9
M15			Describe how responses were processed and/or synthesised. *Include qualitative analyses of free-text responses (for example, thematic, content or cluster analysis) and/or quantitative analytical methods, if used.*	9
M16			Describe any piloting of the study materials and/or survey instruments. *Include how many individuals piloted the study materials, the rationale for the selection of those individuals, any changes made as a result and whether their responses were used in the calculation of the final consensus. If no pilot was conducted, this should be stated.*	8
M17			If applicable, describe how feedback was provided to panellists at the end of each consensus step or meeting. *State whether feedback was quantitative (for example, approval rates per topic/item) and/or qualitative (for example, comments, or lists of approved items), and whether it was anonymised.*	9
M18			State whether anonymity was planned in the study design. Explain where and to whom it was applied and what methods were used to guarantee anonymity.	7
M19			State if the steering committee was involved in the decisions made by the consensus panel. *For example, whether the steering committee or those managing consensus also had voting rights.*	8
M20		Participation	Describe any incentives used to encourage responses or participation in the consensus process. *For example, were invitations to participate reiterated, or were participants reimbursed for their time.*	8
M21			Describe any adaptations to make the surveys/meetings more accessible. *For example, the languages in which the surveys/meetings were conducted and whether translations or plain language summaries were available.*	8
R1		Results	State when the consensus exercise was conducted. List the date of initiation and the time taken to complete each consensus step, analysis, and any extensions or delays in the analysis.	10
R2			Explain any deviations from the study protocol, and why these were necessary. *For example, addition of panel members during the exercise, number of consensus steps, stopping criteria; report the step(s) in which this occurred.*	8

Item No.		Section	Checklist Item (*help text*)	Page No.
R3			For each step, report quantitative (number of panellists, response rate) and qualitative (relevant socio-demographics) data to describe the participating panellists.	10-15
R4			Report the outcome of the consensus process as qualitative (for example, aggregated themes from comments) and/or quantitative (for example, summary statistics, score means, medians and/or ranges) data.	13-15
R5			List any items or topics that were modified or removed during the consensus process. Include why and when in the process they were modified or removed.	16-17
D1		Discussion	Discuss the methodological strengths and limitations of the consensus exercise. *Include factors that may have impacted the decisions (for example, response rates, representativeness of the panel, potential for feedback during consensus to bias responses, potential impact of any non-anonymised interactions).*	19-22
D2			Discuss whether the recommendations are consistent with any pre-existing literature and, if not, propose reasons why this process may have arrived at alternative conclusions.	19-24
O1		Other information	List any endorsing organisations involved and their role.	1, 6
O2			State any potential conflicts of interests, including among those directing the consensus study and panellists. Describe how conflicts of interest were managed.	n/a
O3			State any funding received and the role of the funder. *Specify, for example, any funder involvement in the study concept/design, participation in the steering committee, conducting the consensus process, funding of any medical writing support. This could be disclosed in the methods or in the relevant transparency section of the manuscript. Where a funder did not play a role in the process or influence the decisions reached, this should be specified.*	n/a

From: PLoS Med 21(1): e1004326. https://doi.org/10.1371/journal.pmed.1004326 For more information see: https://www.ismpp.org/accord

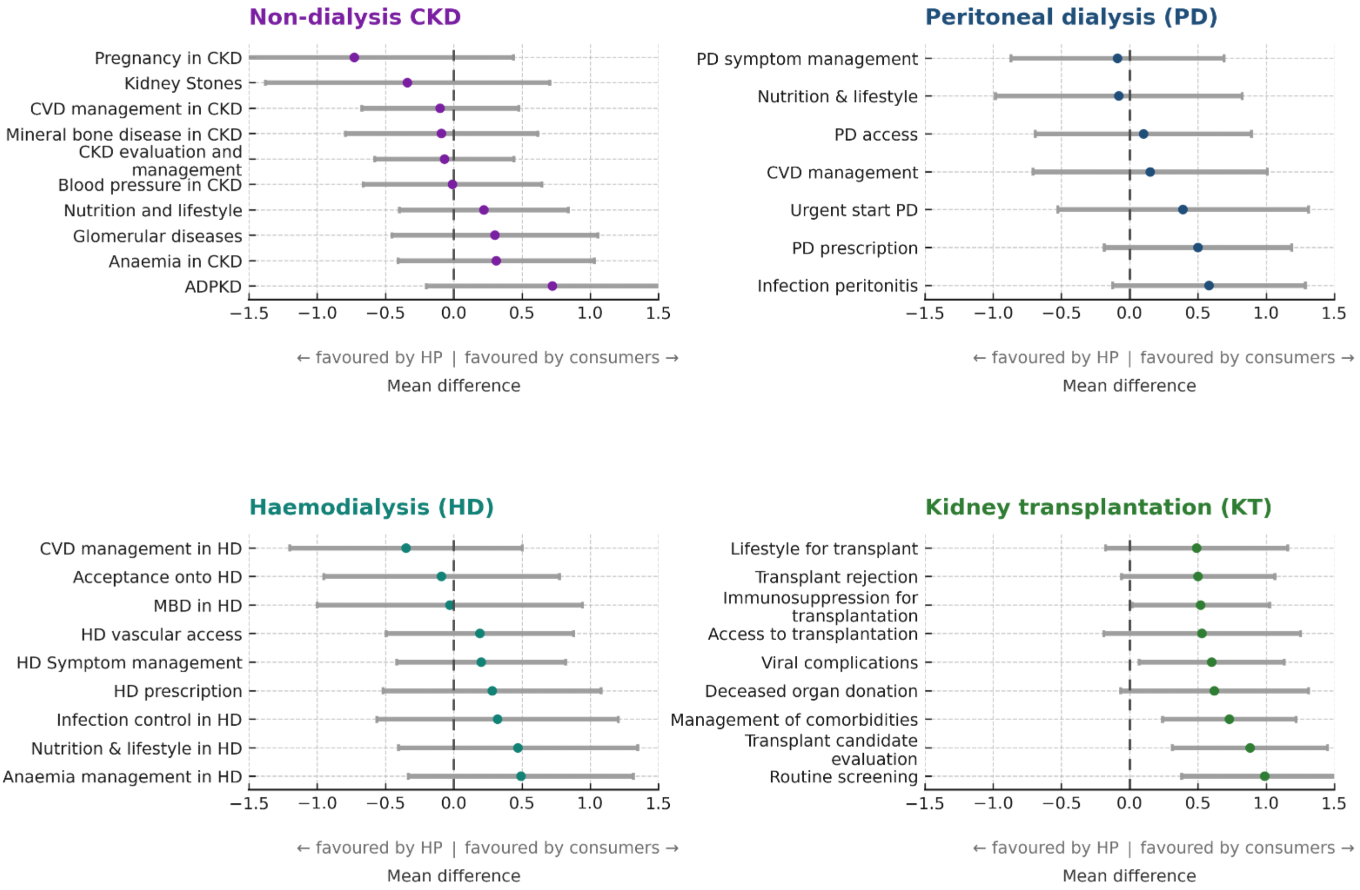

Figure S1. Rd 1 mean Likert scale difference, consumer and health professionals. *Four forest plots show subtopic priorities for Non-dialysis CKD (purple), Peritoneal dialysis (PD) (blue), Haemodialysis (HD) (teal), and Kidney transplantation (KT) (green). Within each panel, rows are ordered from most negative to most positive mean difference in Likert ratings (Consumers − HP).

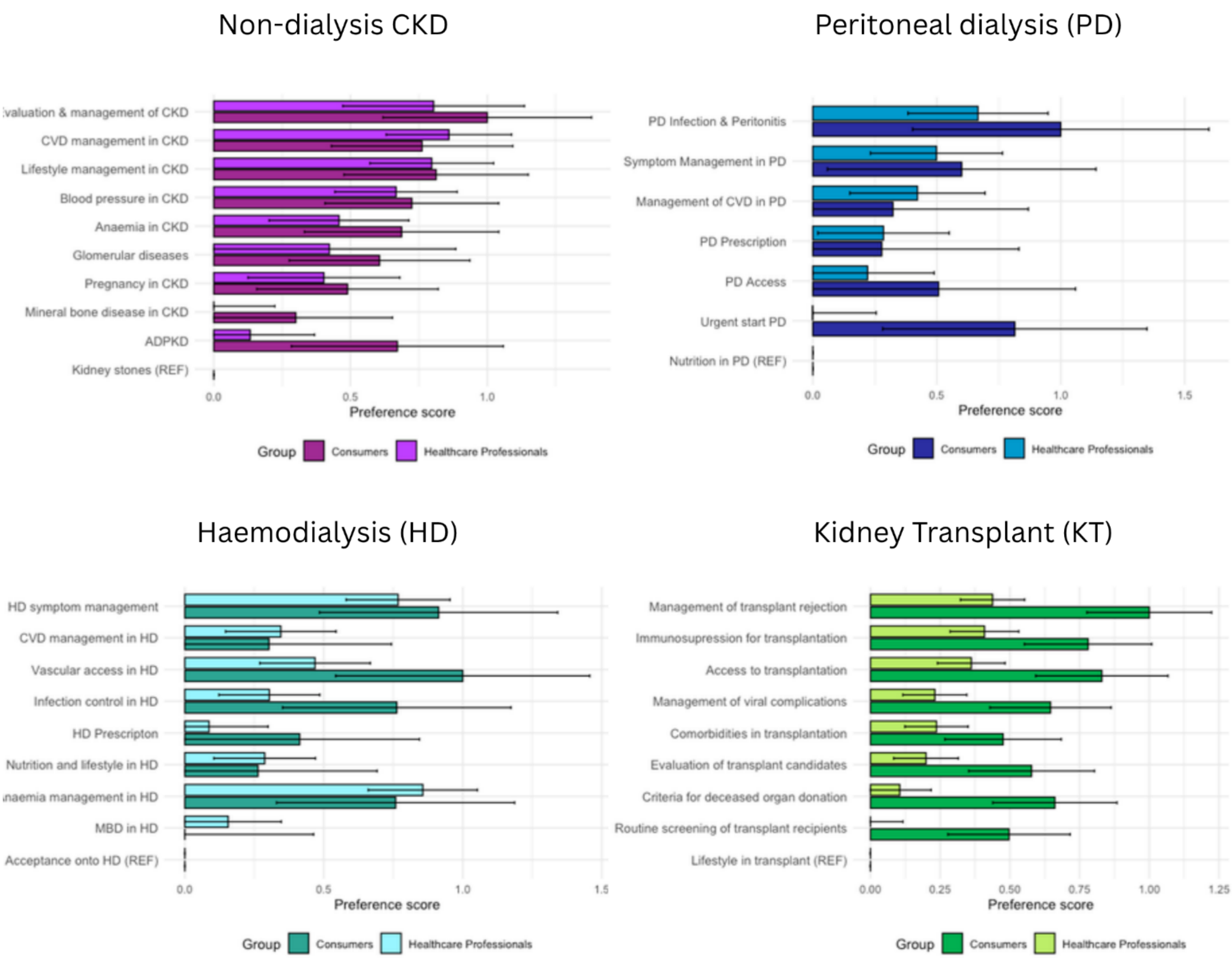

Figure S2. Rd 1 BWS analysis by groups consumer and health professionals.

*Preference scores represent relative topic importance (BSW), error bars show within-group variability (95% confidence interval), and the reference (REF) value indicates the lowest ranked topic. BP = Blood pressure, CKD = chronic kidney disease.

Table S2. Summary statistics for round 1 Likert scores

Module	Broad Topic	n	Mean	SD	% 7-9
**Non-dialysis CKD**	**Evaluation & management of CKD**	81	8.09	1.11	93%
	**CVD management in CKD**	83	7.99	1.14	92%
	**BP management in CKD**	82	7.93	1.27	88%
	**Nutrition & lifestyle in CKD**	83	7.84	1.35	82%
	**Anaemia in CKD**	80	7.50	1.59	75%
	Glomerular diseases	78	7.27	1.57	71%
	Pregnancy in CKD	81	7.14	2.00	74%
	Mineral bone disease in CKD	81	6.98	1.48	65%
	ADPKD	78	6.85	1.72	63%
	Kidney stones	81	6.51	1.83	56%
**Peritoneal Dialysis (PD)**	**Management of Infection & Peritonitis**	49	7.98	1.41	88%
	**Management of Symptoms in PD**	50	7.74	1.31	86%
	**Prescription**	50	7.70	1.18	82%
	**Access to PD**	49	7.67	1.20	86%
	**Management of CVD**	49	7.61	1.29	82%
	Lifestyle & Nutrition for PD	50	7.48	1.40	78%
	Urgent Start PD	47	7.34	1.36	69%
**Haemodialysis (HD)**	**CVD management in HD**	67	7.96	1.33	89%
	**HD prescription**	68	7.85	1.16	92%
	**HD Symptom management**	66	7.83	1.18	88%
	**HD vascular access**	64	7.84	1.24	85%
	**Acceptance onto HD**	68	7.57	1.37	78%
	Infection control in HD	66	7.48	1.38	75%
	Nutrition & lifestyle in HD	66	7.42	1.44	73%
	Anaemia management in HD	67	7.18	1.58	69%
	MBD in HD	67	7.10	1.53	72%
**Transplant**	**Management of transplant rejection**	61	8.36	1.05	96%
	**Immunosuppression for transplantation**	61	8.21	1.18	94%
	**Management of comorbidities**	61	8.08	1.08	89%
	**Management of viral complications**	61	7.93	1.09	87%
	Evaluation of transplant candidates	62	7.84	1.26	83%
	Criteria for deceased organ donation	61	7.79	1.36	79%
	**Access to transplantation**	61	7.66	1.46	82%
	Routine screening	62	7.63	1.36	80%
	Lifestyle for transplant	62	7.45	1.44	71%

**This table summarises mean importance ratings (1 = least important to 9 = critically important) for broad guideline topics within each group, ordered by mean rating from highest to lowest. "% 7-9" is the agreement (%) and refers to the proportion of respondents rating the topic ≥7 (i.e., important to critically important). IQR = interquartile range; SD = standard deviation; n = number of respondents.*

Table S3. Summary statistics for round 2 Non-dialysis CKD Likert scores

Guideline topic	Guideline subtopic	Mean	SD	n	% 7–9
Anaemia	**Diagnosis and evaluation of anaemia**	**7.09**	**1.82**	**74**	**66.2%**
	Monitoring response to treatment and side effects	6.98	1.79	74	64.9%
	Evaluation of iron status	6.78	1.78	74	63.6%
	Patient education and shared decision-making	6.68	2.03	73	50.0%
	Blood transfusions	6.55	1.95	73	61.5%
	Nutritional management and iron therapy	6.53	1.97	73	56.9%
	Erythropoiesis-stimulating agents (ESAs)	6.23	2.22	74	50.0%
Blood Pressure	**Medication management of BP**	**7.16**	**1.72**	**85**	**75.3%**
	**Lowering BP through lifestyle changes**	**7.08**	**1.74**	**85**	**65.7%**
	Accurate measurement of BP	6.80	2.12	85	65.9%
	BP targets	6.68	2.09	85	62.1%
	Dietary adjustments for BP	6.59	2.07	85	60.6%
Cardiovascular Disease	**Cardiovascular disease risk assessment**	**7.00**	**2.02**	**93**	**54.4%**
	Renin-angiotensin system (RAS) inhibition	6.73	2.04	91	48.5%
	Coronary artery disease (CAD)	6.71	1.88	92	63.6%
	Lipids and triglycerides	6.69	1.86	91	60.6%
	Antiplatelet and anticoagulant therapy	6.65	2.02	92	56.9%
	Renovascular disease (RVD)	6.63	2.04	92	61.2%
	Heart failure management	6.57	2.27	92	54.5%
Evaluation and Management	**Treatment to prevent progression and complications**	**7.51**	**2.00**	**105**	**73.2%**
	**Education and self-management**	**7.31**	**1.88**	**105**	**70.5%**
	**Defining and evaluating non-dialysis CKD**	**7.16**	**1.91**	**107**	**70.1%**
	**Models of care for non-dialysis CKD**	**7.02**	**2.10**	**105**	**68.6%**
	Cardiovascular Health in CKD	6.93	2.06	107	63.6%
Lifestyle and Diet	**Dietary patterns for non-dialysis CKD**	**7.25**	**1.58**	**96**	**74.0%**
	**Lifestyle and exercise**	**7.01**	**1.59**	**97**	**70.1%**
	Micronutrients	6.92	1.67	96	57.6%
	Weight management	6.83	1.87	96	66.7%
	Management of acidosis	6.80	1.84	96	56.1%
	Nutrition assessment and support	6.71	1.90	96	53.0%
	Macronutrient and energy intake	6.68	1.74	96	51.5%

**Bolded fields were advanced to round 3*

Table S4. Summary statistics for round 2 PD Likert scores

Guideline topic	Guideline subtopic	Mean	SD	n	% 7–9
**Cardiovascular disease**	**Pharmacological interventions for fluid overload in PD**	**7.32**	1.35	25	**76%**
	**Antiplatelets and anticoagulants in PD**	**7.00**	**1.97**	**26**	**67.7%**
	Monitoring residual renal function	6.88	1.58	26	53.8%
	Dialysate solution for cardiovascular disease protection	6.88	2.29	25	40.0%
	Blood pressure management in PD	6.77	1.55	26	65.4%
	Diet quality in PD for cardiovascular disease management	6.76	1.86	25	72%
	Cardiac testing in PD	6.67	2.23	27	48.2%
	Secondary prevention of CVD events in PD	6.65	1.69	26	53.8%
	Monitoring surrogate measures of cardiovascular disease	6.62	2.22	26	42.3%
	Lipid and triglyceride management	6.54	1.99	26	50.0%
	Determining risk of cardiac death	6.19	1.88	26	38.5%
**Infection and peritonitis**	**Management of volume overload**	**7.38**	1.82	26	**75.0%**
	Treatment of catheter site infections	7.35	1.88	26	62.5%
	**Screening and preventing infections**	**7.31**	1.56	26	**75.0%**
	**Prophylactic antibiotics and antibiotic treatment of peritonitis**	**7.00**	1.82	26	**73.1%**
	Diagnosis of infections	6.96	1.56	26	75.0%
	Catheter placement and care	6.94	1.34	26	75.0%
	Catheter management during peritonitis	6.88	1.55	26	62.5%
	**Dialysis technique**	**6.85**	1.87	26	**87.5%**
**PD access**	Complications and management	7.12	1.83	26	53.8%
	Catheter care and management	6.96	1.89	26	46.2%
	Catheter selection and placement	6.58	1.84	26	38.5%
	Vascular access for PD	6.46	1.78	26	42.3%
**PD prescription**	**Ensuring dialysis adequacy**	**7.38**	1.69	26	**69.2%**
	**Maintenance of residual kidney function**	**7.27**	1.61	26	**65.4%**
	Shared decision-making and improving PD quality	7.12	1.58	26	61.5%
	PD schedule and incremental PD	7.08	1.30	26	59.2%
	Managing membrane dysfunction	6.69	1.51	26	50.0%
**Symptom management**	**Common symptoms and management**	**7.58**	1.31	26	**76.9%**
	**Identification, monitoring, and individualised care**	**7.15**	1.38	26	**73.1%**
	**Education and shared decision-making**	**7.15**	1.46	26	**69.2%**

**Bolded fields were advanced to round 3*

Table S5. Summary statistics for round 2 HD Likert scores

Guideline topic	Guideline subtopic	Mean	SD	n	% 7–9
**Cardiovascular disease**	**Volume control and management of fluid overload in HD**	**7.46**	1.96	41	**78.1%**
	**Blood pressure management in HD**	**7.34**	1.51	41	**75.6%**
	Diabetes management for CVD in HD	7.27	1.93	41	58.5%
	Treatment of secondary hyperparathyroidism	6.85	1.99	41	51.2%
	Dialysate solutions for CVD protection	6.44	2.26	39	48.7%
	Lipid and triglyceride management in HD	6.20	2.33	41	41.5%
**HD prescription**	**HD schedule**	**7.61**	1.59	41	**73.2%**
	**Adverse event management**	**7.24**	1.57	41	**65.9%**
	Dialysis modality selection	7.17	1.71	41	56.0%
	**Incremental HD**	**7.05**	1.85	41	**65.9%**
	Exercise in HD	7.00	1.98	41	56.1%
	Membrane choice and treatment adequacy	6.66	2.28	41	48.8%
	Dialysate and water quality	6.61	2.22	41	51.2%
	Blood chemistry and fluid management	6.59	2.13	41	56.1%
	Anticoagulation management	6.58	2.06	40	45.0%
**Infection control**	**Planning and preparation of vascular access (VA)**	**6.90**	2.05	41	**65.9%**
	Hand hygiene and equipment	6.90	1.95	40	52.5%
	Isolation and cohort practices for blood-borne viruses	6.67	2.05	40	57.5%
	Patient care practices	6.67	1.86	40	57.5%
	Water quality	6.50	2.31	40	52.5%
	Surveillance screening and vaccinations	6.38	1.97	40	45.0%
**Symptom management**	**Targeted management of common symptoms**	**7.22**	1.87	41	**68.3%**
	Patient education and shared decision-making	7.07	1.49	41	61.0%
	**Psychosocial support in HD**	**7.05**	1.78	41	**65.9%**
	Models of care	6.93	1.79	41	58.5%
	Identification and monitoring of care	6.71	1.73	41	51.2%
**Vascular access**	**Placement and creation of vascular access**	**7.34**	1.65	41	**70.1%**
	Complication management	7.18	1.84	40	62.5%
	**Type of vascular access**	**7.27**	1.81	41	**65.9%**
	Vascular access use and care	7.07	1.72	41	58.5%

**Bolded fields were advanced to round 3*

Table S6. Summary statistics for round 2 Transplant Likert scores

Guideline topic	Guideline subtopic	Mean	SD	n	% 7–9
Access to transplantation	**Eligibility and selection**	**7.80**	1.16	44	**88.6%**
	**Disparities in access**	**7.66**	1.22	44	**81.8%**
	**Pre-operative management**	**7.57**	1.53	44	**79.6%**
	Ethical considerations	7.30	1.67	44	72.7%
	Living vs. deceased donor	7.05	1.69	44	59.1%
Comorbidities	**Management of Obesity**	**7.78**	1.17	45	**84.4%**
	**Managing and preventing cancer**	**7.73**	1.47	45	**84.4%**
	**Management of cardiovascular disease (CVD)**	**7.53**	**1.54**	**45**	**80.0%**
	**Management of diabetes**	**7.53**	**1.65**	**45**	**82.2%**
	Management of Psychological Conditions	7.57	1.35	45	71.1%
	Managing mineral bone disease (MBD)	7.33	1.33	45	77.8%
	Management of haematological complications	7.16	1.49	45	64.4%
	Management of Pulmonary Disease	7.00	1.78	44	68.2%
	Management of hyperuricemia and gout	6.98	1.80	44	56.8%
Immunosuppression	**Monitoring and management of immunosuppression**	**7.95**	1.15	42	**88.1%**
	**Management of side effects**	**7.90**	1.32	41	**87.8%**
	**Induction immunosuppression**	**7.81**	1.42	42	**76.2%**
	**Patient education**	**7.69**	1.64	42	**76.2%**
	**Initial maintenance**	**7.52**	1.83	42	**76.2%**
KT Rejection	**Long term immunosuppressive strategies and pre-emptive treatment**	**7.80**	1.64	45	**75.6%**
	**Treatment of chronic rejection**	**7.80**	1.38	45	**82.2%**
	**Post rejection monitoring**	**7.62**	1.65	45	**77.8%**
	Treatment of acute rejection	7.60	1.55	45	68.9%
	Detection of transplant rejection	7.49	1.60	45	77.8%
	Identify high-risk of transplant rejection	7.32	1.77	44	63.6%
	Imaging to detect transplant rejection	7.20	1.89	45	60.0%
Viral complications	**Ongoing monitoring of immunosuppressive therapy**	**7.80**	1.25	44	**84.1%**
	**Prevention and management of bacterial and fungal infections**	**7.75**	1.19	44	**84.1%**
	Antiviral prophylaxis	**7.67**	1.32	43	**72.1%**
	**Viral monitoring and management**	**7.50**	1.47	44	**81.8%**
	Vaccinations	7.27	1.92	44	75.0%

**Bolded fields were advanced to round 3. "Ongoing monitoring of immunosuppressive therapy" and "Monitoring and management of immunosuppression" were combined into one topic for round 3.*

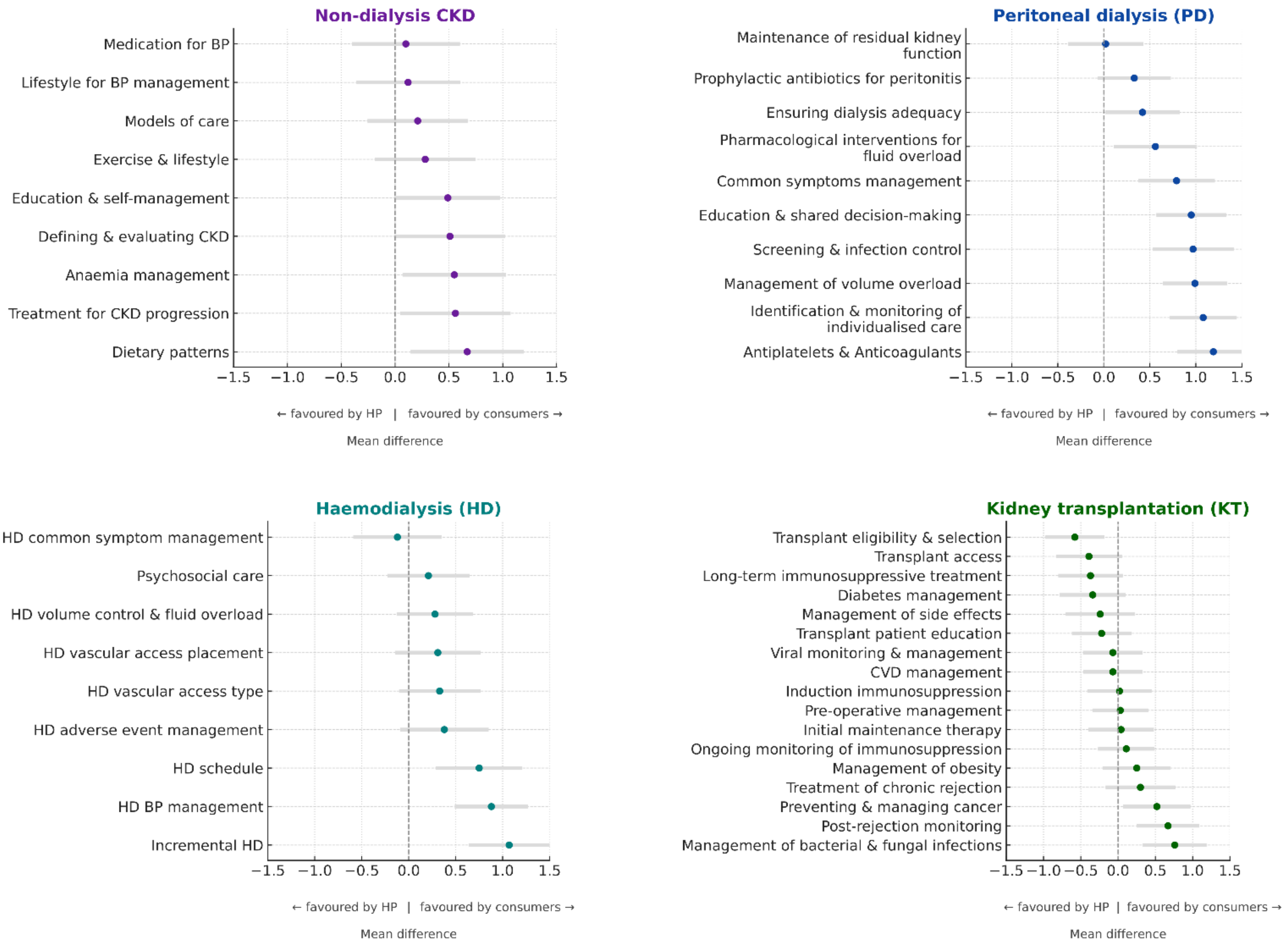

Figure S3. Rd 3 mean Likert scale difference, consumer and health professionals. ******Four forest plots show subtopic priorities for* ***Non-dialysis CKD*** *(purple),* ***Peritoneal dialysis (PD)*** *(blue),* ***Haemodialysis (HD)*** *(teal), and* ***Kidney transplantation (KT)*** *(green). Within each panel, rows are ordered from* ***most negative*** *to* ***most positive*** *mean difference in Rd3 Likert ratings (Consumers − HP).*

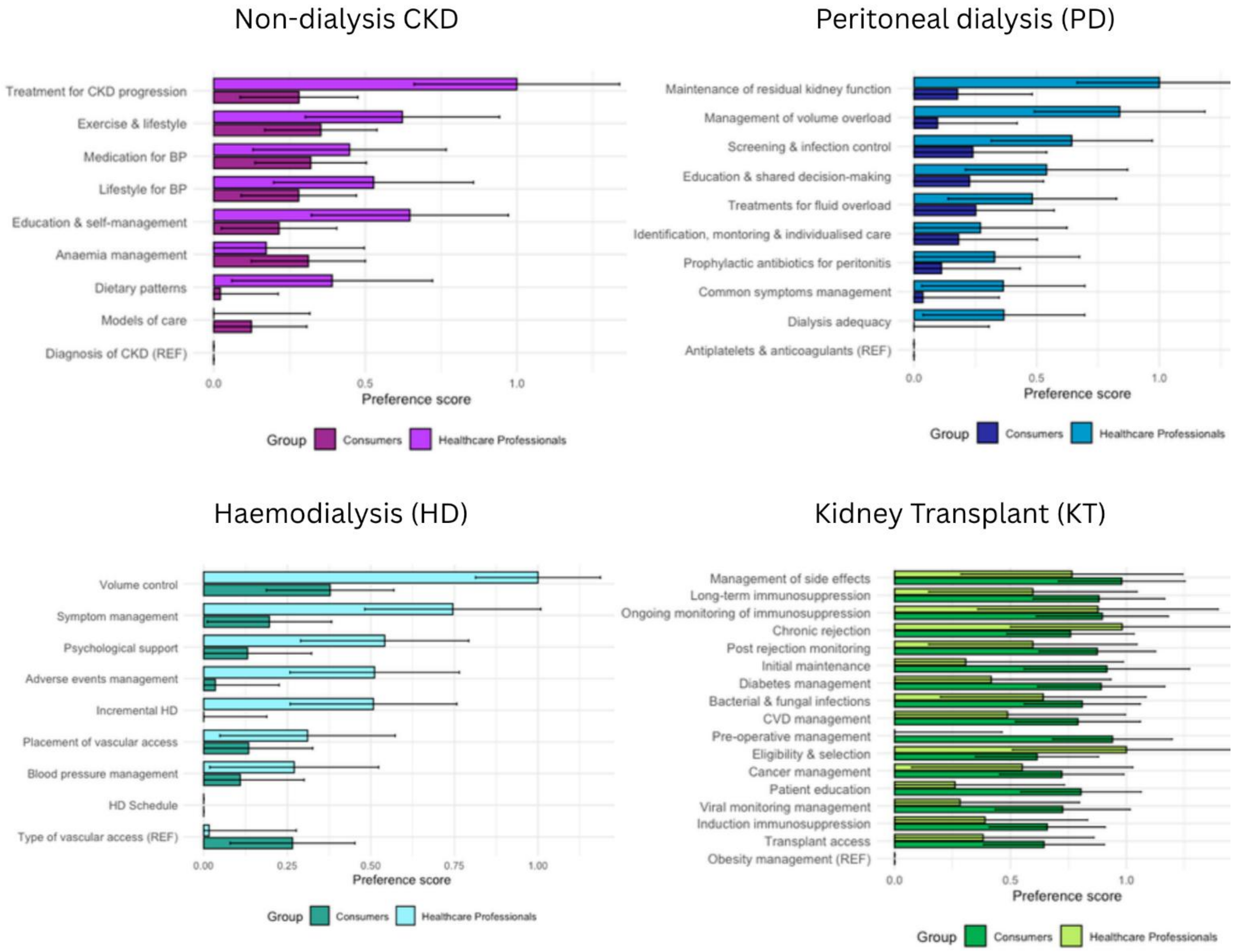

Figure S4. Rd 3 BWS by module: **consumers vs health professionals**.
Points show relative preference score (±95% CI);* ***REF* *= lowest-ranked item per module. REF items:* ***Non-dialysis CKD****—Diagnosis of CKD;* ***PD****—Antiplatelets/anticoagulants;* ***HD****—Creation/type of vascular access;* ***KT****—Management of obesity. Ordered by combined consumer priorities.*
Abbreviations: *BP, blood pressure; CKD, chronic kidney disease.*

Table S7. Summary statistics for round 3 guideline subtopics by module

Module	Sub-topic	n	Mean	SD	% 7- 9
**Non-dialysis CKD**	Education & self-management	415	6.48	2.46	59
	Exercise & lifestyle for CKD	412	6.41	2.43	57
	Treatment for CKD progression	419	6.37	2.53	56
	CKD models of care	417	6.24	2.44	54
	Lifestyle for BP management	415	6.13	2.51	51
	Defining & evaluating CKD	417	6.08	2.64	55
	Anaemia management	416	6.06	2.48	53
	Dietary patterns for CKD	414	6.04	2.74	53
	Medications for BP	425	5.88	2.67	50
**Peritoneal Dialysis (PD)**	Education & shared decision making	92	7.36	1.98	74
	Management of volume overload	95	7.20	1.84	68
	Maintenance of residual kidney function	93	7.19	2.10	77
	Prophylactic antibiotics for peritonitis	91	7.03	2.02	70
	Common symptoms management	96	7.02	2.15	68
	Ensuring dialysis adequacy	93	6.95	2.06	69
	Identification & monitoring of individualised care	90	6.84	1.93	62
	Pharmacological interventions for fluid overload	89	6.78	2.33	65
	Screening & infection control	95	6.77	2.29	67
	Antiplatelets & Anticoagulants in PD	94	6.52	2.09	57
**Haemodialysis (HD)**	HD volume control & fluid overload	158	7.15	2.12	75
	Incremental HD	161	7.00	2.28	69
	Psychosocial care	162	7.03	2.30	69
	HD adverse event management	161	6.89	2.47	72
	HD common symptom management	166	6.84	2.46	64
	HD schedule	157	6.83	2.40	67
	HD BP management	164	7.02	2.10	66
	HD vascular access type	162	6.81	2.30	64
	HD vascular access placement	165	6.67	2.39	61
**Transplant**	CVD management	155	7.21	2.20	75
	Post-rejection monitoring	158	7.11	2.17	72
	Treatment of chronic rejection	156	7.03	2.37	69
	Ongoing monitoring of immunosuppression	153	7.03	2.04	66
	Transplant eligibility & selection	154	6.97	2.27	68
	Viral monitoring & management	156	6.97	2.29	69
	Cancer management	154	6.93	2.35	68
	Transplant patient education	154	6.92	2.19	65
	Pre-operative management	158	6.92	2.14	68
	Transplant access	158	6.85	2.36	61
	Long-term immunosuppressive treatment	160	6.83	2.27	63
	Bacterial & fungal infections	157	6.83	2.24	62
	Induction immunosuppression	155	6.72	2.36	65
	Initial maintenance therapy	153	6.70	2.41	61
	Management of side effects	156	6.65	2.53	65
	Diabetes management	159	6.65	2.41	64
	Management of obesity	159	6.58	2.48	61

**This table summarises mean importance ratings (1 = least important to 9 = critically important) round 3 subtopics within each module, ordered by mean rating from highest to lowest. "% 7-9" is the agreement (%) and refers to the proportion of respondents rating the topic ≥7 (i.e., important to critically important). IQR = interquartile range; SD = standard deviation; n = number of respondents.*
